# TB-SERS analyzer: Analysis tool for tuberculosis prediction based on Raman spectroscopy with machine learning and convolutional neural network

**DOI:** 10.1371/journal.pcbi.1014397

**Published:** 2026-07-14

**Authors:** Jukgarin Eisiri, Chadatan Juntagran, Kanwara Trisakul, Benjawan Kaewseekhao, Noppadon Nuntawong, Chakchai So-In, Kiatichai Faksri

**Affiliations:** 1 Multidisciplinary Department, Graduate School, Khon Kaen University, Khon Kaen, Thailand; 2 Research and Diagnostic Center for Emerging Infectious Diseases (RCEID), Khon Kaen University, Khon Kaen, Thailand; 3 Department of Microbiology, Faculty of Medicine, Khon Kaen University, Khon Kaen, Thailand; 4 National Electronics and Computer Technology Center (NECTEC), National Science and Technology Development Agency (NSTDA), Pathum Thani, Thailand; 5 Department of Computer Science, College of Computing, Khon Kaen University, Khon Kaen, Thailand; University of Minho, PORTUGAL

## Abstract

Raman spectroscopy (RS) and surface-enhanced Raman spectroscopy (SERS) are promising technologies that have been applied across various fields, including clinical diagnostics. In the context of tuberculosis (TB) diagnosis, RS/SERS offers significant potential for rapid, non-invasive, and sensitive biomolecular detection. However, no software currently exists that is specifically designed to analyze RS/SERS data for TB diagnosis. Our goal is to develop such a tool by integrating machine learning (ML) and a one-dimensional convolutional neural network (1D-CNN) into a user-friendly graphical user interface (GUI). We introduce TB-SERS Analyzer, a Python-based tool with a GUI for tuberculosis prediction using SERS data. A reference database of 1,000 plasma samples (500 IGRA-positive, 500 IGRA-negative) was established using the interferon-gamma release assay (IGRA). TB-SERS Analyzer allows users to input spectral data and automatically generate TB diagnostic reports. ML and 1D-CNN models were trained and optimized via five-fold stratified cross-validation. We evaluated seven algorithms to identify the most effective method for TB classification. The 1D-CNN model achieved 82.00% sensitivity and 76.00% specificity in the validation set (n = 200). In a blinded external test (n = 20), the model maintained 80.00% sensitivity with 100% specificity. The software comprises four integrated modules: (1) patient data extraction, (2) data preparation, (3) ML and 1D-CNN analysis, and (4) diagnostic report generation. TB-SERS Analyzer demonstrated high efficiency in TB screening, delivering results in under 10 seconds per sample. TB-SERS Analyzer is an effective and accessible tool for TB screening, combining RS/SERS technologies with ML and 1D-CNN models. The software is freely available on GitHub at: https://github.com/jkeisiri/TB-SERS-Analyzer.

## Author summary

Tuberculosis (TB) remains one of the world’s leading infectious diseases, affecting millions of people annually and posing a major global public health challenge. Early screening and diagnosis are critical for controlling disease transmission and improving patient outcomes; however, many conventional diagnostic methods require specialized equipment, trained personnel, and time-consuming laboratory procedures. Raman spectroscopy has emerged as a promising analytical technology capable of rapidly detecting disease-associated biochemical changes in biological samples without extensive sample preparation. Nevertheless, translating complex spectral data into clinically meaningful diagnostic information remains challenging and often requires advanced computational approaches. To address this need, we developed ***TB-SERS Analyzer***, a user-friendly software platform for tuberculosis prediction based on Raman spectroscopy data. The software integrates spectral preprocessing, data visualization, artificial intelligence-based classification, and automated report generation into a single workflow, enabling users to obtain diagnostic predictions with minimal technical expertise. Using a large collection of blood plasma samples, we evaluated multiple machine-learning and deep-learning approaches and identified a deep learning model that demonstrated promising classification performance for TB detection. By simplifying Raman spectral analysis, TB-SERS Analyzer enhances the accessibility of Raman-based diagnostics and may facilitate future research, clinical translation, and the development of Raman spectroscopy-based approaches for tuberculosis screening.

## Introduction

Tuberculosis (TB) remains a significant global public health challenge with profound impacts on individuals, economies, and societies worldwide. This disease is caused by the bacterium *Mycobacterium tuberculosis* (*Mtb*), which spreads through the air when an infected person coughs, sneezes, or spits [1]. In 2022, approximately 1.3 million people worldwide succumbed to TB [1]. Approximately, one in four people globally is infected with the bacterium that causes TB. In latent tuberculosis infection (LTBI), the host immune system continuously responds to *Mtb* antigen despite the absence of apparent clinical symptoms. While those with LTBI cannot spread the bacteria, active TB disease will eventually develop in about 5–10% of those individuals [1]. Additionally, the gold standard test to diagnose LTBI is unestablished [2]. Present screening tests for LTBI are time-consuming, laborious, and expensive, such as the interferon gamma release assay (IGRA), tuberculin skin test (TST), and *Mycobacterium tuberculosis* antigen-based skin tests (TBST) [3,4]. IGRA cannot distinguish between active TB and LTBI, and the sensitivity and specificity remain low [5,6]. The End-TB strategy of the World Health Organization [7] cannot be achieved without more effective screening tools for TB.

To fill this gap, Raman spectroscopy (RS) and surface-enhanced Raman spectroscopy (SERS) techniques can be applied to improve the effectiveness of TB screening. The RS/SERS approach is considered a reliable advancement to analyze chemical components in pathogenic bacteria [8]. This technique can detect COVID-19 in human serum [9] and discriminate pathogenic *Mycobacterium* species from nonpathogenic ones [10]. The method has also been validated for TB detection, accurately discriminating *Mtb*-positive sputum from *Mtb*-negative sputum, suggesting the successful differentiation of sputum samples with or without *Mtb* strains using machine learning (ML) analysis of RS/SERS data, achieving an accuracy of 94.32% [11]. Our study shows that RS/SERS can distinguish active TB and LTBI by serum-protein fingerprinting among active TB, LTBI, early clearance, and healthy control (HC) [12]. Our recent study, which used 1,000 samples from IGRA-positives (IGP) and IGRA-negatives (IGN), found promising diagnostic models with high accuracy when combining RS/SERS and ML [13].

Recent advances in ML and deep learning (DL) have further improved TB prediction and classification across biosensing and clinical data platforms [14]. In Raman- and SERS-based TB studies, spectral classification has increasingly benefited from supervised ML methods such as support vector machines (SVM), random forest (RF), k-nearest neighbors (KNN), and partial least squares-discriminant analysis (PLS-DA), as well as DL architectures including convolutional neural networks (CNNs), which can automatically learn discriminative spectral features with minimal manual preprocessing [15]. These approaches have shown promising performance in distinguishing TB-related samples from controls and in identifying subtle biomolecular signatures associated with infection. Beyond spectroscopy, ML/DL has also been widely applied to TB prediction using chest X-ray images, computed tomography, clinical metadata, and microbiological data, often achieving high sensitivity and specificity in classification tasks [16]. However, despite this growing body of work, most existing studies have focused on model development rather than on providing an integrated, user-friendly software environment for routine TB screening and report generation. This highlights the need for a dedicated platform that combines robust ML/DL algorithms with practical usability for RS/SERS-based TB diagnosis.

Effective analysis and management of complex biological data are especially important for TB diagnostic models based on RS/SERS spectra. However, despite advances in Raman spectroscopy hardware, dedicated software tools for comprehensive TB analysis remain unavailable. Therefore, this study aimed to develop software to generate diagnostic reports for TB using spectral data from RS and SERS. The software was designed to analyze a dataset of 1,000 cases, comprising both IGP and IGN samples from our previous study [13]. We aim to deliver accurate and user-friendly diagnostic outputs, particularly an intuitive graphical user interface (GUI) that simplifies the diagnostic workflow and enables users to navigate the software easily. By leveraging RS/SERS technologies, the software may enhance both the precision and efficiency of TB diagnosis. Furthermore, it can establish a foundational platform for future applications in other medical domains that employ Raman and SERS techniques.

## Design and implementation

### Ethics statement

The study protocol was approved by the Khon Kaen University Ethics Committee for Human Research (approval number HE664037). The requirement for informed consent was waived by the committee, as anonymized leftover specimens from the biobank and datasets retrieved from previously published studies were used.

### Software architecture and implementation

The TB-SERS Analyzer was developed using Python 3.8 and is currently distributed as a precompiled for Microsoft Windows. While the source code is platform-independent and can, in principle, be compiled on other operating systems, official precompiled versions for non-Windows platforms are not yet provided. This may limit immediate usability in non-Windows environments. The software employs PyQt5 for GUI application [17] and SQLite 3 for local data storage. The core engine processes IGRA samples using scikit-learn and TensorFlow/Keras [18], while reports are generated via the FPDF package [19]. The software was developed and tested on a laptop equipped with an Intel Core i5-10300H (2.50 GHz), 16 GB RAM, and an NVIDIA GeForce GTX 1650. However, to ensure accessibility in resource-limited settings, the recommended minimum hardware requirements estimated based on the operational demands of TensorFlow and PyQt5 are a multi-core processor and 4 GB of RAM. The TB-SERS Analyzer operates entirely offline and requires no programming expertise, allowing for one-click analysis and report generation.

### Data preparation and preprocessing

The Raman spectroscopy input used in this study comprises 1,000 datasets of SERS-mapped spectra from human plasma specimens, as previously described in [13] and illustrated in **Fig A** in S1 Appendix. The dataset consists of two clinical groups: 500 IGP and 500 IGN cases. Each dataset was generated from a 7 × 7 Raman mapping grid (49 spectra per sample), covering a spectral range of 620–1,720 cm^-1^ with 1,011 data points per spectrum. The instrument settings for the Raman spectroscopic measurements followed the parameters detailed in the previously published protocol [13] and all spectra underwent preprocessing including noise reduction, artifact removal, and background fluorescence correction. This included: (i) removal of cosmic ray spikes using a modified Z-score method with a threshold of 8, where detected spikes were replaced by the median of neighboring points; (ii) outlier detection and removal based on principal component analysis (PCA), where spectra were projected onto the first five principal components and filtered using robust Mahalanobis distance (Minimum Covariance Determinant) with a 90% confidence threshold; (iii) baseline correction using a third-order polynomial fitting approach to reduce background fluorescence; (iv) shifting each spectrum such that its minimum intensity was set to zero; (v) normalization using standard normal variate (SNV); and (vi) after preprocessing (**Fig B** in S1 Appendix), spectra from each sample were averaged to obtain a representative spectrum for downstream analysis.

All spectra used for model development and evaluation were acquired using the same instrument and processed using an identical preprocessing pipeline to ensure consistency. Furthermore, all preprocessing steps were implemented in Python, and the corresponding code is publicly available (https://github.com/jkeisiri/TB-SERS-Analyzer/tree/main/analysis) to support reproducibility.

### Model optimization

To develop an effective classification model, we compared several supervised ML algorithms and a one-dimensional convolutional neural network (1D-CNN), including the design and optimization of appropriate model structures. We evaluated seven widely used and validated algorithms: linear discriminant analysis (LDA), logistic regression (LR), RF, SVM, KNN, extreme gradient boosting (XGB), and 1D-CNN. All ML algorithms were implemented using the scikit-learn library, except for the 1D-CNN model, which was developed using the Sequential API in Keras within the TensorFlow framework. For conventional machine learning models, hyperparameter optimization was performed using GridSearchCV with stratified five-fold cross-validation (CV), using the area under the receiver operating characteristic curve (ROC-AUC) as the scoring metric. The best-performing model from the grid search was then used to generate cross-validated out-of-fold predictions using the same CV scheme, enabling unbiased estimation of model performance. The specific optimized parameters for each ML candidate (LR, LDA, SVM, KNN, RF, and XGB) are provided in **Table** **A** in S1 Appendix. For the 1D-CNN model, the architecture including convolutional blocks, batch normalization, Leaky Rectified Linear Unit (Leaky ReLU), and pooling layers, was optimized through a manual tuning process (**Tables B–F** in S1 Appendix). Model performance was evaluated using 5-fold stratified CV. In each fold, four subsets were used for training, and one subset was held out for validation. The validation subset was used to monitor training convergence and implement early stopping, as well as to evaluate model performance. This approach ensures that each sample is evaluated in an out-of-fold manner on the training set, while reducing the risk of overfitting and improving the generalizability of the model.

**Fig 1:**
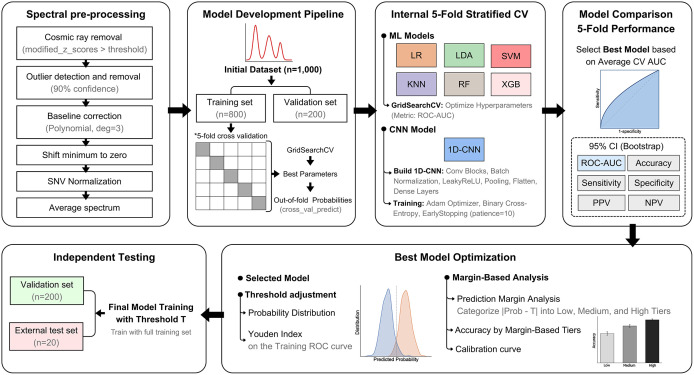
Schematic workflow of the model development and evaluation pipeline for the TB-SERS Analyzer. The pipeline comprises several sequential stages: (1) Spectral preprocessing, including cosmic ray removal, outlier detection, baseline correction, and SNV normalization to generate representative average spectra. (2) Model Development, where the initial dataset (n = 1,000) is partitioned into training (n = 800) and validation (n = 200) sets. (3) Internal 5-Fold Stratified CV, utilized for hyperparameter optimization via GridSearchCV for six machine learning (ML) models and architecture tuning for the 1D-CNN. (4) Model Comparison, where the best-performing model is selected based on average CV ROC-AUC and 95% Confidence Intervals (Bootstrap). (5) Best Model Optimization, involving threshold adjustment using the Youden Index and margin-based analysis. (6) Independent Testing, where the final optimized model is validated using both the internal validation set and an external test set (n = 20). *Note: During 1D-CNN training within the cross-validation and final retraining phases, 20% of the training data was reserved as a validation set for monitoring convergence and early stopping.*

Following optimization, the models were ranked based on their average CV ROC-AUC and 95% confidence intervals (calculated via bootstrapping). The best-performing model was then integrated into the TB-SERS Analyzer pipeline (Fig 1). To further enhance diagnostic precision and dependability, this selected model underwent additional optimization, including threshold adjustment using the Youden Index, ensuring high sensitivity and specificity for tuberculosis screening and margin-based analysis.

### One-dimensional CNN architecture

**Fig 2:**
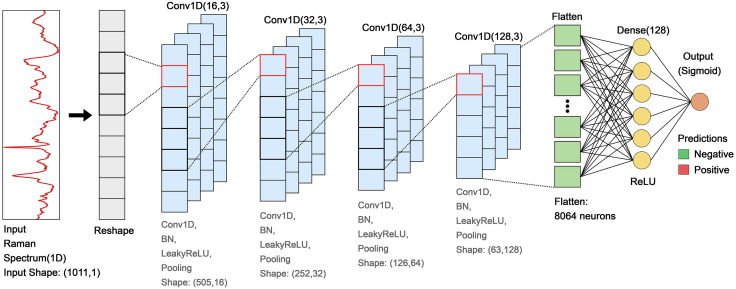
Architecture of the proposed 1D-CNN model for Raman spectral classification. The model comprises four convolutional layers, each with a kernel size of 3 and progressively increasing filter sizes: 16, 32, 64, and 128. Each convolutional layer is followed by batch normalization, a Leaky Rectified Linear Unit (Leaky ReLU) activation function, and average pooling with a pool size of 2. The resulting feature maps are flattened and passed through a fully connected dense layer containing 128 neurons. The final output layer consists of a single neuron with a sigmoid activation function for binary classification. The model is optimized using the Adam optimizer with a learning rate of 0.0001 and trained with early stopping to prevent overfitting.

The proposed 1D-CNN for Raman spectral classification includes four convolutional layers (Fig 2) with kernel size 3, stride 1, and ‘same’ padding to preserve input length. The number of filters increases across layers: 16, 32, 64, and 128. Each layer is followed by batch normalization, Leaky ReLU activation, and average pooling (pool size 2) to reduce dimensionality while retaining spectral features. Extracted features are flattened and passed through a dense layer with 128 neurons, followed by a sigmoid-activated output neuron for binary classification. The model is trained for 100 epochs using the Adam optimizer (learning rate 0.0001) with a batch size of 16. Early stopping (patience 10) prevents overfitting. The loss function is binary cross-entropy, and classification accuracy is employed to measure performance.

### Performance evaluation and final classification algorithms

The diagnostic performance of the candidate algorithms was rigorously evaluated following the model development pipeline (Fig 1). The initial dataset (n = 1,000) was strictly partitioned into a training set (n = 800) and an independent validation set (n = 200). To ensure the integrity of the evaluation, all model development including hyperparameter tuning via 5-fold stratified cross-validation and 1D-CNN architecture optimization was conducted exclusively using the training set. The validation set was reserved solely as an unseen dataset for the final performance assessment to verify the model ability to generalize to new data.

Standard evaluation metrics were employed to quantify model performance, including sensitivity (Eq 1), specificity (Eq 2), accuracy (Eq 3), positive predictive value (PPV) (Eq 4), and negative predictive value (NPV) (Eq 5). Furthermore, the ROC-AUC were calculated. To ensure statistical reliability and assess model generalizability, 95% confidence intervals (CI) for all metrics were determined using a bootstrapping method (1,000 iterations).

The TB-SERS Analyzer prioritized the algorithm that achieved the highest consolidated AUC. The final optimized model performance was further confirmed through independent testing on the validation set and an external test set (n = 20). This multi-stage validation ensures that the selected algorithm is robust and provides dependable TB classification within the SERS spectral analysis framework.

The following equations were used to compute the classification performance metrics:


$$\begin{array}{c} \text{ Sensitivity or TPR }=\frac{TP}{\left(TP+FN\right)} \end{array}$$ (1)

$$\begin{array}{c} \text{ Specificity }=\;\frac{TN}{\left(TN+FP\right)} \end{array}$$ (2)

$$\begin{array}{c} \text{ Accuracy}=\frac{\left(TP+TN\right)}{\left(TP+FN+FP+TN\right)} \end{array}$$ (3)

$$\begin{array}{c} \;\;\text{PPV}\;\text{or }\text{Precision}\;=\;\frac{TP}{\left(TP+FP\right)} \end{array}$$ (4)

$$\begin{array}{c} \;\text{NPV}\;=\;\frac{TN}{\left(TN+FN\right)} \end{array}$$ (5)

where TP, TN, FP, and FN denote true positives, true negatives, false positives, and false negatives, respectively.

### Biological significance and spectral feature analysis

To enhance the interpretability of the computational models and identify the biological signatures driving the classification, a systematic sub-region performance analysis was conducted. The fingerprint region (620–1720) cm^-1^) was partitioned into six discrete Raman shift intervals: (620–800), (800–1000), (1000–1200), (1200–1400), (1400–1600), and (1600–1720) cm^-1^.

The diagnostic performance (ROC-AUC) was evaluated independently for each sub-region [20] using the best-performing model architecture, validated through 5-fold cross-validation on the training set. Following this, prominent Raman peaks within these high-performing regions representing key biomolecular markers such as lipids, proteins, or nucleic acids [21], were selected for further statistical validation. The intensity distributions of these specific peaks were compared between the IGP and IGN groups using the Mann-Whitney U test. This approach transitions from purely algorithmic classification to a feature-based biological analysis, correlating the model response with the underlying biochemical variations associated with different clinical and pathological conditions.

### Threshold determination and margin-based prediction analysis

To facilitate interpretation of model outputs, predicted probability scores for IGP and IGN classifications were generated. These scores represent the model-predicted probability of each class based on the input spectral signatures.

The generation of these probabilities was based on the best-performing model architecture identified during the model selection process. The optimal screening threshold for the TB-SERS Analyzer was determined using the training set through internal 5-fold stratified CV. In this process, out-of-fold probability scores were collected across the training dataset. The Youden Index [22] was then applied to the resulting ROC curve to identify the threshold that maximizes the balance between sensitivity and specificity.

To further characterize model output behavior, the absolute margin between the predicted probability and the optimal classification threshold (|predicted probability − threshold|) was calculated [23]. These values were derived from out-of-fold predictions during CV, ensuring that each prediction was generated from data not seen during model training. This margin represents the relative distance of each prediction from the decision boundary and was used as a proxy measure for ranking model predictions based on their distance from the decision boundary.

Based on the distribution of margin values, predictions were stratified into three tiers (low, medium, and high) using the 30th and 70th percentiles. This stratification provides a relative ranking of predictions, where higher-margin cases are more clearly separated from the decision boundary, while lower-margin cases represent borderline predictions that may require cautious interpretation.

To further examine this stratification, classification performance was evaluated independently within each tier. In addition, calibration plots were generated to assess the agreement between predicted probabilities and observed IGRA outcomes. To improve probability calibration, isotonic regression was applied [23]. Model performance was compared before and after calibration to evaluate improvements in calibration performance. Calibration curves were generated using 10 bins (n = 10; quantile-based).

### Description of TB-SERS Analyzer workflow for individual sample analysis

**Fig 3:**
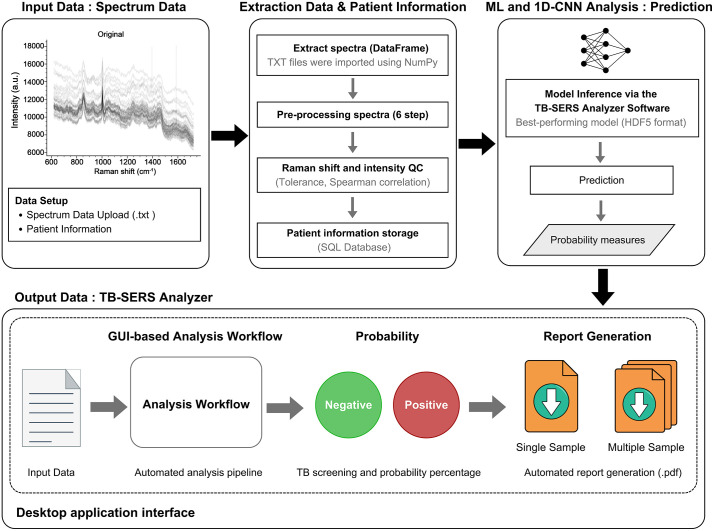
Overall workflow and analysis pipeline of the TB-SERS Analyzer. The workflow consists of four main steps: **“Input Data”** – Users upload spectral data and optionally input patient information for preprocessing. **“Data Extraction”** – After preprocessing the raw data and performing Raman shift and intensity quality control (QC), DataFrame structures are generated containing all processed intensity and Raman shift (cm^-1^) values. Patient information is stored in an SQL database for reference. **“ML and 1D-CNN Analysis”** – The best-performing algorithm, as determined within the TB-SERS Analyzer, is used to predict outcomes for new input data. **“Output Data”** – Diagnostic results are categorized as either single-sample or batch outputs. Prediction results are compiled and exported as a PDF report, which can be saved immediately upon completion of processing.

The process flow for analyzing individual samples using the TB-SERS Analyzer is illustrated in Fig 3. The workflow consists of four main steps:

1) Input Spectrum Data: A spectral data file (in.txt format) is uploaded to the system. Optional patient information associated with the sample can also be entered at this stage.
2) Data Extraction and Patient Information: The uploaded file is read using the NumPy package [24], currently supporting text-based.txt format, as no other file formats were available for evaluation. Preprocessing is then applied. Raman shifts and intensity alignment with the reference model are used to perform quality control check on blinded samples. Raman shift confirmation was performed using the isclose() function from the NumPy package (i.e., numpy.isclose) [24], with an absolute tolerance of 3 cm^*-1*^ to account for minor variations in Raman peak positions. The spectral similarity of Raman intensity profiles is assessed using Spearman correlation (*ρ*) [25]. A warning is issued when ρ falls below the predefined threshold, indicating potentially poor spectral quality or that the sample lies outside the training data distribution, which may compromise prediction reliability. Correlation strength was interpreted based on Chan YH [26] as follows: *ρ* ≥ 0.9 indicates high similarity; *ρ* ≥ 0.8 allows minor variation; *ρ* ≥ 0.7 is moderate but acceptable; and *ρ* < 0.7 requires further review. Final analytical decisions remain user dependent. Processed spectra are stored as Pandas DataFrame [27] structures, with patient data maintained in an SQL database.
3) ML and 1D-CNN Analysis: Preprocessed sample data stored in DataFrame structures were formatted to meet the input requirements of the classification algorithm. The diagnostic prediction is then performed using the best-performing model.
4) Output Data: Upon completion of the analysis, the final prediction probabilities, patient information**,** and diagnostic results are compiled and exported in a.pdf report format.

### External testing of the TB-SERS Analyzer using blinded plasma samples

Twenty blinded plasma samples, each with known IGRA results, were used for external validation of the TB-SERS Analyzer. These samples were collected by the Office of Disease Prevention and Control, Region 7, Khon Kaen, Thailand. Spectral measurements were performed using a Raman spectroscopic system following the protocol previously published by our group [13]. The study protocol for sample collection and analysis was approved by the Human Research Ethics Committee of Khon Kaen University (Approval No. HE664037).

## Results

### Comparative performance of machine Learning algorithms and 1D-CNN for IGRA classification

The diagnostic performance of the seven candidate algorithms was evaluated using 5-fold stratified cross-validation (CV) on the training set (n = 800). The 1D-CNN model demonstrated superior overall discriminative performance, achieving the highest AUC of 0.885 (95% CI: 0.86–0.90) (Table 1). While conventional machine learning models such as XGB (AUC = 0.872) and RF (AUC = 0.865) showed competitive performance, the 1D-CNN maintained a more robust balance across all key metrics, including an accuracy of 77.50% and a sensitivity of 75.50%. Confusion matrices for all models evaluated via cross-validation are provided in **Fig C** in S1 Appendix.

**Table 1:** Performance comparison of the 1D-CNN and other machine learning models for IGRA classification during the training phase (5-fold cross-validation, n = 800).

Model	Model Performance Metrics (5-Fold Cross-Validation, n = 800)						
	Sensitivity (%, 95% CI)	Specificity (%, 95% CI)	Accuracy (%, 95% CI)	PPV (%, 95% CI)	NPV (%, 95% CI)	AUC (95% CI)	DeLong test (*p*-value)*
1D-CNN	**75.50% (0.71-0.80)**	**79.50% (0.76-0.83)**	**77.50% (0.74-0.80)**	**78.65% (0.74-0.83)**	**76.44% (0.72-0.80)**	**0.885 (0.86-0.90)**	**–**
LR	72.50% (0.68-0.77)	75.25% (0.71-0.79)	73.88% (0.71-0.77)	74.55% (0.70-0.79)	73.24% (0.69-0.77)	0.824 (0.79-0.85)	< 0.001
LDA	71.75% (0.67-0.76)	74.00% (0.70-0.78)	72.88% (0.70-0.76)	73.40% (0.69-0.78)	72.37% (0.68-0.77)	0.817 (0.79-0.85)	< 0.001
KNN	68.75% (0.64-0.73)	79.25% (0.75-0.83)	74.00% (0.71-0.77)	76.82% (0.72-0.81)	71.72% (0.67-0.76)	0.838 (0.81-0.87)	< 0.001
SVM	77.75% (0.74-0.82)	77.75% (0.73-0.82)	77.75% (0.75-0.81)	77.75% (0.74-0.82)	77.75% (0.74-0.82)	0.860 (0.83-0.88)	0.005
RF	76.00% (0.72-0.80)	78.50% (0.74-0.82)	77.25% (0.74-0.80)	77.95% (0.74-0.82)	76.59% (0.73-0.81)	0.865 (0.84-0.89)	0.013
XGB	77.00% (0.73-0.81)	80.00% (0.76-0.84)	78.50% (0.76-0.81)	79.38% (0.75-0.83)	77.67% (0.74-0.82)	0.872 (0.85-0.89)	0.124

Note: CI: confidence interval; PPV: positive predictive value; NPV: negative predictive value; AUC: area under the receiver operating characteristic curve; 1D-CNN: one-dimensional convolutional neural network; LR: logistic regression; LDA: linear discriminant analysis; KNN: k-nearest neighbors; SVM: support vector machine; RF: random forest; XGB: extreme gradient boosting. *Statistical significance was determined using the DeLong test compared with the 1D-CNN model. *p*-values < 0.001 indicates strong statistical significance; *p*-values ≥ 0.05 are considered non-significant (ns).

To rigorously validate the superiority of the 1D-CNN, the DeLong test was performed to compare its ROC curve with those of the other algorithms. The statistical analysis revealed that the 1D-CNN significantly outperformed LR, LDA, and KNN (*p* < 0.001), as well as SVM (*p* = 0.005) and RF (*p* = 0.013). Although the performance difference between the 1D-CNN and XGB did not reach statistical significance (*p* = 0.124), the 1D-CNN was ultimately selected as the core architecture for the TB-SERS Analyzer due to its higher AUC.

### Sub-region performance analysis and biomolecular interpretation

To elucidate the biochemical basis of the 1D-CNN classification, a sub-region spectral analysis was performed. As shown in **Fig D.A** in S1 Appendix, the fingerprint region was evaluated across six discrete Raman shift intervals. The sub-region between 1000–1200 cm^-1^ yielded the highest diagnostic performance with an AUC of 0.805. In comparison, the full-spectrum model achieved a higher AUC of 0.885, indicating that while the selected sub-region captures relevant biochemical information, the complete spectral profile provides additional discriminative features for distinguishing between the IGRA result groups.

The average SERS spectra for the IGN and IGP groups (**Fig D.B** in S1 Appendix) revealed several prominent peaks corresponding to major biomolecules. Further statistical investigation was focused on the most discriminative peaks within the high-performing 1000–1200 cm^-1^ region. Significant intensity variations were observed at 1003 cm^-1^ (phenylalanine), 1030 cm^-1^ (proteins/C-C skeletal), 1050 cm^-1^ (C-N and C-C stretching), 1097 cm^-1^ (nucleic acids/ phosphate stretching), and 1139 cm^-1^ (proteins CH_2_ and CH_3_ bending) (**Table G** in S1 Appendix).

Statistical comparison using the Mann-Whitney U test (**Fig D.C** in S1 Appendix) confirmed that these intensity differences were highly significant (*p* < 0.001 for most peaks). For instance, the peak at 1003 cm^-1^ showed a marked decrease in the IGP group, while the peak at 1050 cm^-1^ exhibited an increase (*p* = 0.008). These results demonstrate that the 1D-CNN model decisions are effectively correlated with specific biochemical alterations in plasma, reinforcing the biological validity of the TB-SERS Analyzer.

### Threshold optimization and performance across margin-based prediction tiers in TB-SERS Analyzer

To optimize the practical utility of the best-performing 1D-CNN model, the classification threshold and model output behavior were further evaluated. The optimal threshold was determined using the Youden Index on the training set ROC curve (AUC = 0.885). As shown in **Fig E** in S1 Appendix, a threshold of 0.581 was identified, achieving a sensitivity of 72.00% and specificity of 86.70%. The density distribution of predicted probabilities showed a clear separation between the IGN and IGP groups, although moderate overlap was observed near the decision boundary.

Furthermore, a tiered stratification approach was established based on the margin between the predicted probability and the classification threshold. The data were stratified into low-, medium-, and high-margin tiers using cut-offs of 0.2317 and 0.4176 (**Fig F** in S1 Appendix). Evaluation of this stratification (**Fig G** in S1 Appendix) showed that higher-margin tier was associated with improved classification accuracy. Specifically, in the validation set, the high-margin tier achieved an accuracy of 95.5% (n = 89), whereas the low-margin tier exhibited lower accuracy (48.2%), indicating the presence of borderline predictions.

To assess the reliability of predicted probabilities, isotonic regression was applied for calibration. The calibration plots (**Fig H** in S1 Appendix) demonstrate that, prior to adjustment (black lines), the predicted probabilities deviated from the ideal diagonal. Following calibration (red lines), the predictions aligned more closely with the reference line in both the training and validation sets, indicating improved agreement between predicted probabilities and observed outcomes. Importantly, the overall model performance remained comparable before and after calibration, indicating that probability adjustment did not compromise discriminative ability.

These results demonstrate that the TB-SERS Analyzer provides probabilistic outputs that can be further stratified based on their distance from the decision boundary, enabling more nuanced interpretation of model predictions.

### Graphical user interface and visualization of TB-SERS Analyzer from raw data

TB-SERS Analyzer was developed as a GUI-based tool for direct RS/SERS input data, allowing users to follow a simple diagnostic workflow. Final diagnostic results are exported as PDF files. Users can customize reports by modifying titles and uploading logos.

For single-sample analysis, the report displays results from Raman shift and intensity quality control, the classification result (IGP or IGN), and the prediction probability as calculated by the 1D-CNN model. Results are visualized using a dynamic color indicator: green for IGP and red for IGN. Margin-based prediction tiers are also color-coded: green (high), yellow (medium), and orange (low). For multiple samples, results are presented in tabular form.

The system was evaluated by four blinded plasma samples with complete patient information. TB-SERS Analyzer takes approximately less than 10 seconds to analyze and report per sample (**Table H** in S1 Appendix).

Despite non-programming requirements, users should provide patient information and raw RS/SERS input data. After saving, the input data will be stored in an SQLite database, which maintains sample records and corresponding file paths. The software also supports batch analysis. Additionally, example SERS spectral datasets are provided to facilitate data input, testing, and user-managed data comparison.

### Validation and external blind testing of the TB-SERS Analyzer

Following the selection of the 1D-CNN as the optimal architecture, the model underwent a final retraining phase using the full training dataset (n = 800). To ensure robust convergence and prevent overfitting, a nested 20% validation subset (n = 160) was reserved from this training data specifically for monitoring the learning process and implementing early stopping. The 1D-CNN training (**Fig I** in S1 Appendix) showed steady loss reduction, with the optimal validation loss reached at Epoch 29. Beyond this ‘Best Epoch,’ validation accuracy stabilized at 77.5% while training accuracy continued to rise, indicating the ideal point to prevent overfitting.

**Fig 4:**
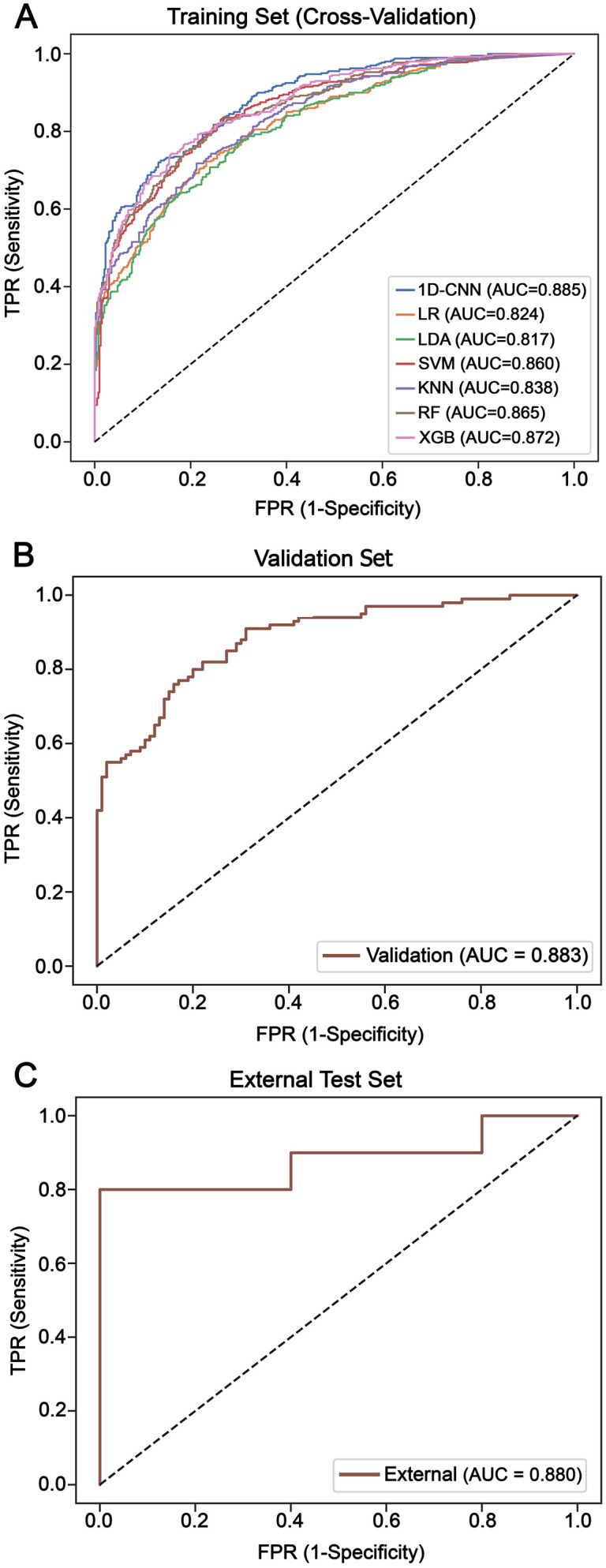
Receiver operating characteristic (ROC) curves for diagnostic model performance and validation. **(A)** Comparison of machine learning and deep learning models on the Training Set using 5-fold cross-validation. The models evaluated include Linear Discriminant Analysis (LDA), Logistic Regression (LR), Random Forest (RF), Support Vector Machine (SVM), K-Nearest Neighbors (KNN), Extreme Gradient Boosting (XGB), and a One-Dimensional Convolutional Neural Network (1D-CNN). The 1D-CNN model demonstrated superior diagnostic performance with an Area Under the Curve (AUC) of 0.885. **(B)** Performance of the best-performing 1D-CNN model on the Validation Set, achieving an AUC of 0.883. **(C)** Performance of the 1D-CNN model on the External Test Set, maintaining high diagnostic robustness with an AUC of 0.880.

The generalization capability of the TB-SERS Analyzer was rigorously validated using two independent datasets: an internal validation set (n = 200) and an external test set (n = 20) comprising blinded plasma samples. As shown in **Fig 4** (ROC Curves), the 1D-CNN model maintained exceptional stability, yielding an AUC of 0.883 for the validation set and 0.880 for the external test set. These results indicate that the model effectively retained its discriminative power when encountering entirely new clinical samples.

**Fig 5:**
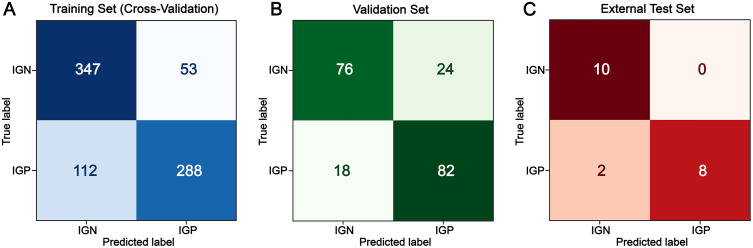
Confusion matrices of the 1D-CNN model for IGN and IGP classification. The diagnostic accuracy of the optimized 1D-CNN model was evaluated across three datasets: (A) the training set (using 5-fold cross-validation), (B) the validation set, and (C) the external test set. The rows represent the true biological labels (ground truth), while the columns represent the labels predicted by the Raman spectroscopic model. Numerical values within each cell indicate the count of samples. The model demonstrated strong generalization, particularly in the external test set, where it correctly classified all 10 IGN samples and 8 out of 10 IGP samples. Darker shading in the diagonal cells signifies higher classification counts for TN and TP, reflecting the robust discriminative power of the Raman fingerprints.

The classification accuracy was further examined through confusion matrices (Fig 5). In the validation set, the analyzer correctly identified 76/100 IGN and 82/100 IGP cases, achieving an overall accuracy of 79.00%. More importantly, during the external blind test which simulated a real-world clinical workflow, the analyzer achieved a high accuracy of 90.00%, with perfect specificity (100.00%) for the IGN group and 80.00% sensitivity for the IGP group (Table 2).

**Table 2:** Performance evaluation of the TB-SERS Analyzer on the validation set (n = 200) and the blinded external test set (n = 20).

Model	Diagnostic Performance Metrics					
	Sensitivity (%, 95% CI)	Specificity (%, 95% CI)	Accuracy (%, 95% CI)	PPV (%, 95% CI)	NPV (%, 95% CI)	AUC (95% CI)
Validation Set (n = 200)	82.00% (0.74-0.89)	76.00% (0.68-0.85)	79.00% (0.73-0.84)	77.36% (0.69-0.85)	80.85% (0.73-0.88)	0.883 (0.83-0.92)
External test set (n = 20)	80.00% (0.50-1.00)	100.00% (1.00-1.00)	90.00% (0.75-1.00)	100.00% (1.00-1.00)	83.33% (0.56-1.00)	0.880 (0.68-1.00)

Note: Data are presented as value (95% confidence interval). All metrics except AUC are expressed as percentages. Abbreviations: AUC, area under the receiver operating characteristic curve; CI, confidence interval; NPV, negative predictive value; PPV, positive predictive value. The validation set represents 20% of the internal data not used during model training, while the external test set consists of 20 independent blinded samples.

Detailed analysis of individual blinded samples (**Table I** in S1 Appendix) demonstrates the practical utility of the margin-based prediction tiers. For instance, sample Blinded #1 was correctly classified as IGRA-negative with a high probability (99.94%) and a substantial prediction margin (0.4182), resulting in a High-tier assignment. Conversely, samples with probabilities closer to the decision threshold (e.g., Blinded #2) were flagged as Low-tier, highlighting cases where the model output is less definitive and may require closer clinical correlation.

### Availability and future directions

The TB-SERS Analyzer source code is publicly available at https://github.com/jkeisiri/TB-SERS-Analyzer. The software was developed in Python and is distributed as a precompiled application for Microsoft Windows. While the source code is platform-independent and can be compiled or executed on other operating systems, official precompiled versions for non-Windows platforms are not currently provided, which may limit immediate usability in those environments. The program requires Python (v3.8 or higher) and the following dependencies: *fpdf2* (v2.8.5), *matplotlib* (v3.10.8), *pandas* (v2.3.3), pybaselines (v1.2.1), *pyinstaller* (v6.13.0), *PyQt5* (v5.15.11), *scikit-learn* (v1.7.2), and *TensorFlow* (v2.13.0). Detailed installation and usage instructions are provided in the accompanying user manual and the GitHub README file.

The software’s limitations present opportunities for improvement. First, increasing sample diversity (e.g., age or collection sites) will enhance the model to learn the spectral patterns associated with LTBI, improving accuracy and robustness. Biological variability among groups may impact diagnostic performance, and more representative training samples including disease controls (e.g., active TB and HC), can help mitigate this. Importantly, the absence of a true gold standard for determining latent TB status remains a key challenge. In this study, LTBI classification relies solely on immunological responses, as measured by the IGRA. However, IGRA cannot distinguish active TB from LTBI and may exhibit variable performance across different populations [28]. Consequently, IGRA-derived labels may introduce a degree of misclassification into the training data. The model should therefore be interpreted as learning patterns associated with IGRA-defined immune responses rather than definitive clinical TB status. Future work will focus on validation using alternative reference standards and more diverse clinical cohorts. Second, further optimization of ML or DL could improve LTBI detection. While IGRA serves as our reference standard, it has known limitations (e.g., limited use in low- and middle-income countries [LMICs], low diagnostic accuracy for active TB, and operational and cost constraints) in clinical TB diagnosis [5]. Third, TB-SERS Analyzer’s GUI could be upgraded to a web-based platform, allowing users to access it remotely. Lastly, scaling the software to accommodate larger datasets and integrating more advanced data management tools will increase its efficiency and utility in both clinical and research settings.

Looking ahead, TB-SERS Analyzer could evolve into a broader diagnostic platform. Its analytical engine can be adapted to detect other diseases, including cancer, viral infections, or other bacterial infections [29–31]. The system could also serve as a real-time clinical reporting tool. Ideally, the codebase must remain flexible, extensible, and capable of supporting various diagnostic models through ML and DL frameworks, and these frameworks can identify specific spectral signatures.

## Conclusions

We have developed a spectral analysis software platform—TB-SERS Analyzer—that integrates ML and 1D-CNN combined RS/SERS datasets derived from human plasma. The tool features a user-friendly graphical user interface, and a straightforward protocol designed to support and simplify the TB screening process. The software supports both single-sample and batch analyses through an intuitive, click-and-run interface. Upon completion of data processing, diagnostic results are automatically generated in PDF report format. Importantly, the tool demonstrated consistency and reproducibility when analyzing data acquired from different RS/SERS datasets. Validation using blind samples confirmed the tool’s applicability and diagnostic potential. TB-SERS Analyzer represents a novel application of machine learning and deep learning technologies for the prediction of LTBI, offering a rapid, accessible, and scalable alternative to traditional diagnostic methods.

## Supporting information

S1 Appendix Supplementary figures (A-I) and tables (A-I). (PDF)
