# Supplementary material for: TB-SERS analyzer: Analysis tool for tuberculosis prediction based on Raman spectroscopy with machine learning and convolutional neural network
Source: PLoS Comput Biol. 2026 Jul 14;22(7):e1014397. doi: 10.1371/journal.pcbi.1014397 (PMC13379083; doi:10.1371/journal.pcbi.1014397)
Supplement: S1 Appendix — (PDF) [file pcbi.1014397.s001.pdf]

Supporting information for
TB-SERS Analyzer: Analysis tool for tuberculosis prediction based on Raman spectroscopy with machine learning and convolutional neural network

List of abbreviations

AUC	area under the curve
IGRA	interferon-gamma release assay
KNN	k-nearest neighbors
LDA	linear discriminant analysis
LR	logistic regression
ML	machine learning
RF	random forest
ROC	receiver operating characteristic curve
SERS	surface-enhanced Raman spectroscopy
SVM	support vector machine
TB	tuberculosis
XGB	extreme gradient boosting
1D-CNN	1D convolutional neural network

Supplementary Figures

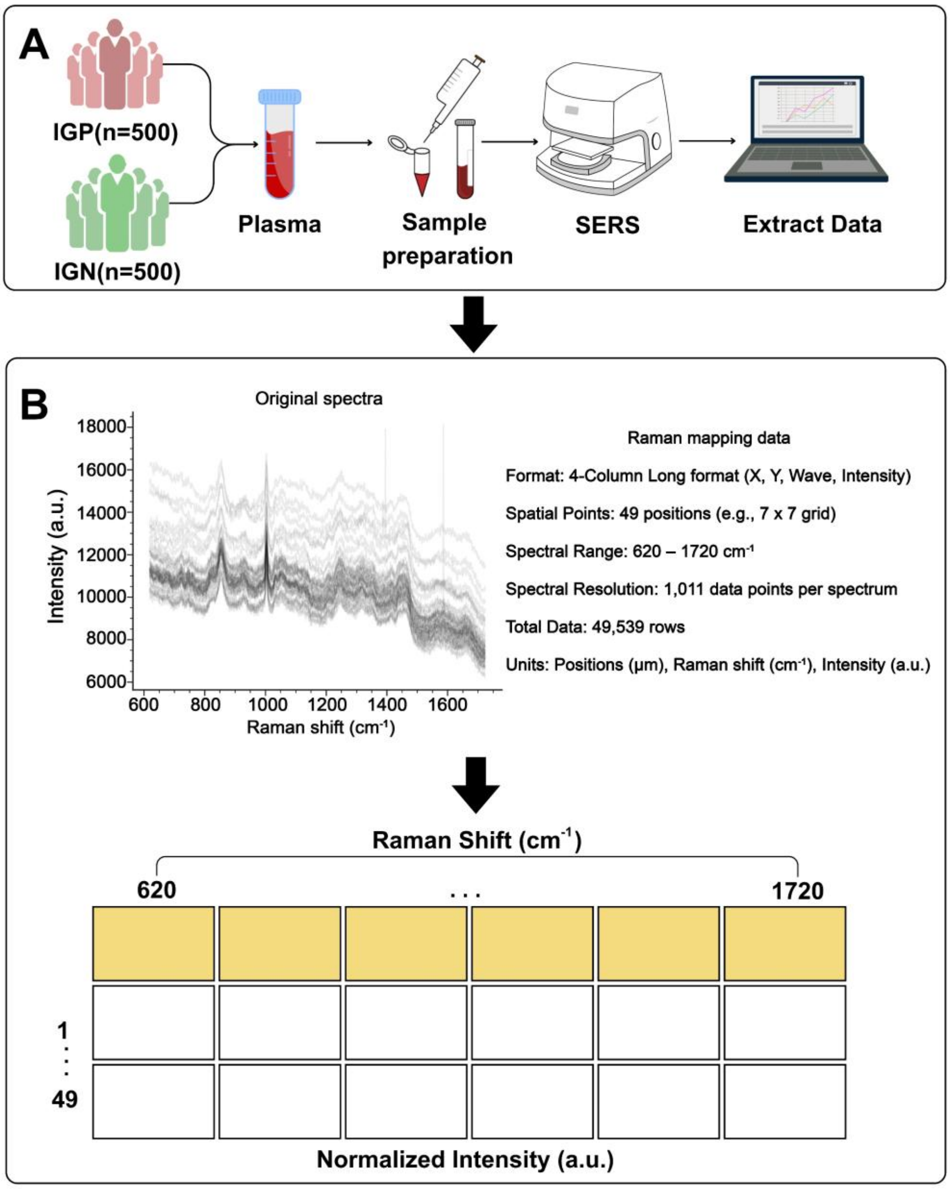

Fig A. Overview of the SERS dataset and data structure. (A) Data Acquisition workflow: The study utilized 1,000 human plasma specimens, categorized into IGRA-positive (IGP, n = 500) and IGRA-negative (IGN, n = 500) groups. (B) Spectral Data Structure: Each specimen was characterized by a 7 × 7 Raman mapping grid, yielding 49 individual spectra. Each spectrum contains 1,011 data points within the fingerprint region of 620–1,720 cm^{-1}, specifically optimized for tuberculosis screening. The raw data (4-column format) was preprocessed to extract normalized relative intensities (a.u.) for machine learning model development. *Note: a.u. = arbitrary unit; IGN = IGRA-negative; IGP = IGRA-positive.*

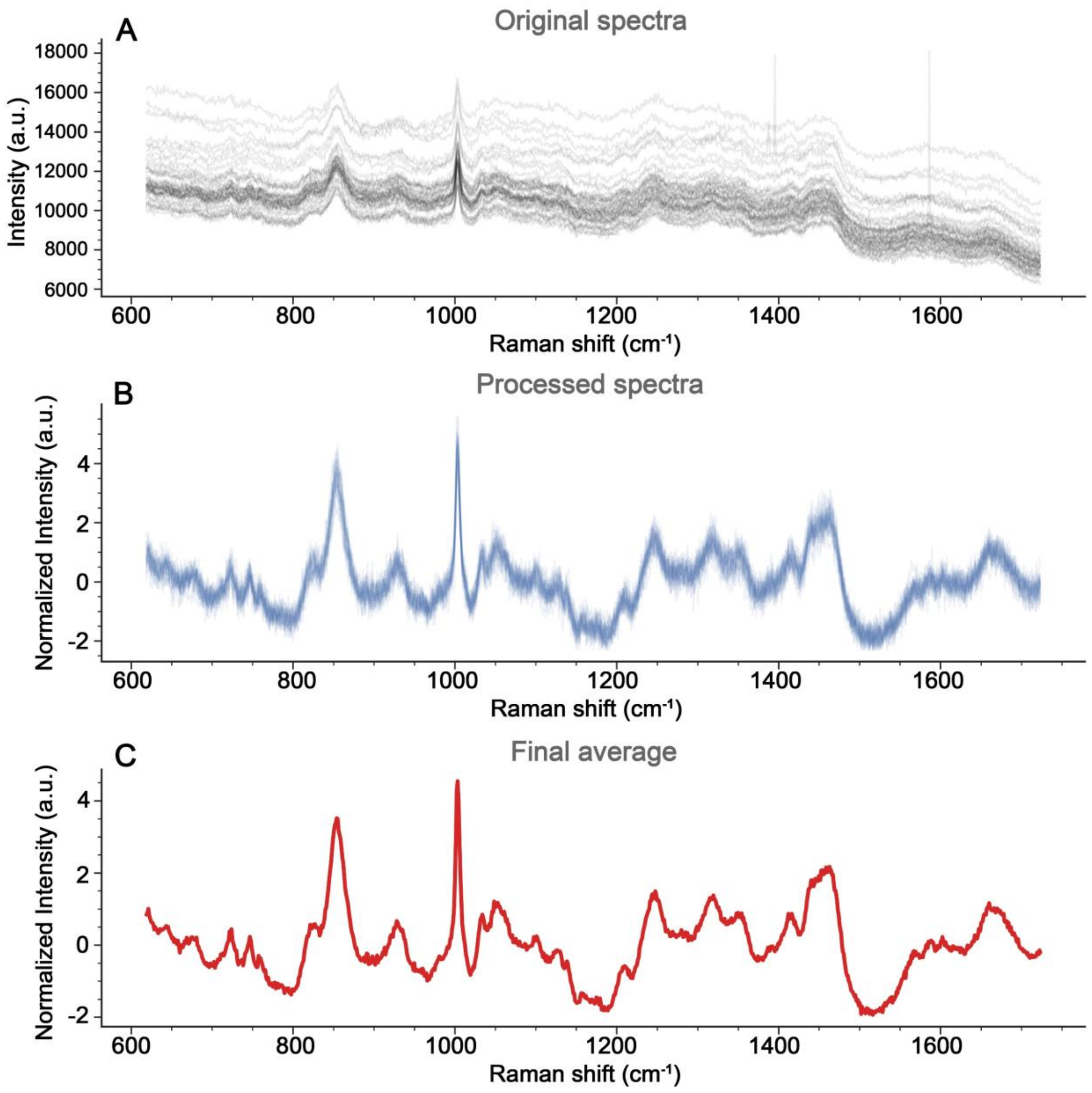

Fig B. Spectral preprocessing workflow and result visualization. The figure illustrates the systematic stages of data refinement to enhance signal quality. (A) Original spectra: Raw SERS data as acquired from the mapping grid, exhibiting cosmic ray artifacts and fluorescence background. (B) Processed spectra: Representative output after the sequential application of Cosmic ray removal (via modified Z-score), Outlier detection (90% confidence interval), Baseline correction (3rd-degree polynomial), and SNV normalization. This stage ensures that the spectral data are comparable and free from non-chemical interference. (C) Final average: The remaining high-quality spectra following outlier removal from the initial 49 mapping points are averaged into a single representative profile. This consolidated spectrum serves as a robust chemical signature.

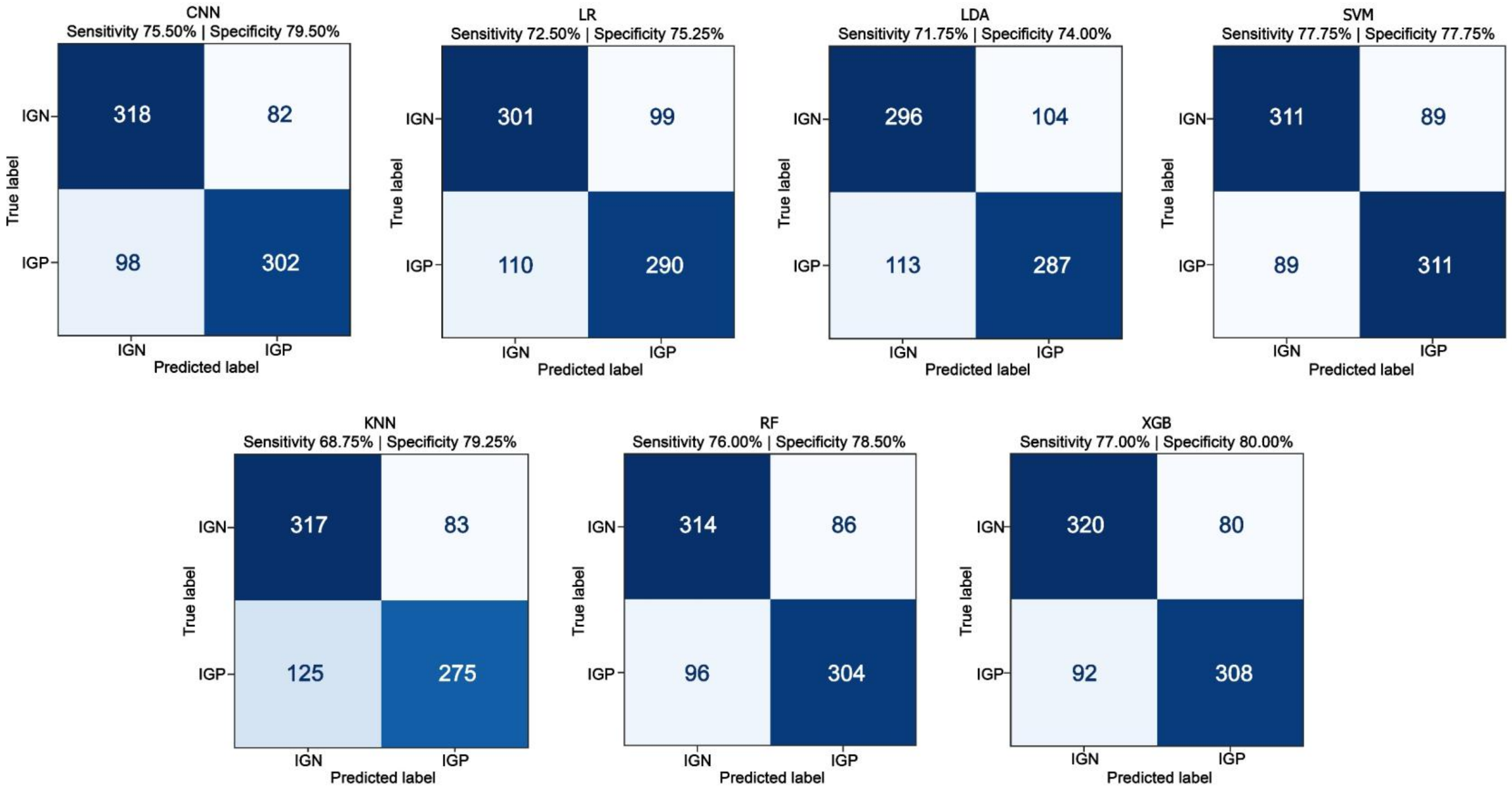

Fig C. Confusion matrices of machine learning models evaluated via 5-fold cross-validation. The confusion matrices illustrate the classification performance for distinguishing between IGRA-negative (IGN) and IGRA-positive (IGP) samples within the training set. To ensure the generalizability of the models and mitigate overfitting, a 5-fold cross-validation approach was employed. These matrices represent the aggregated results across all folds after hyperparameter tuning, serving as a primary metric for selecting the best performing model for final evaluation.

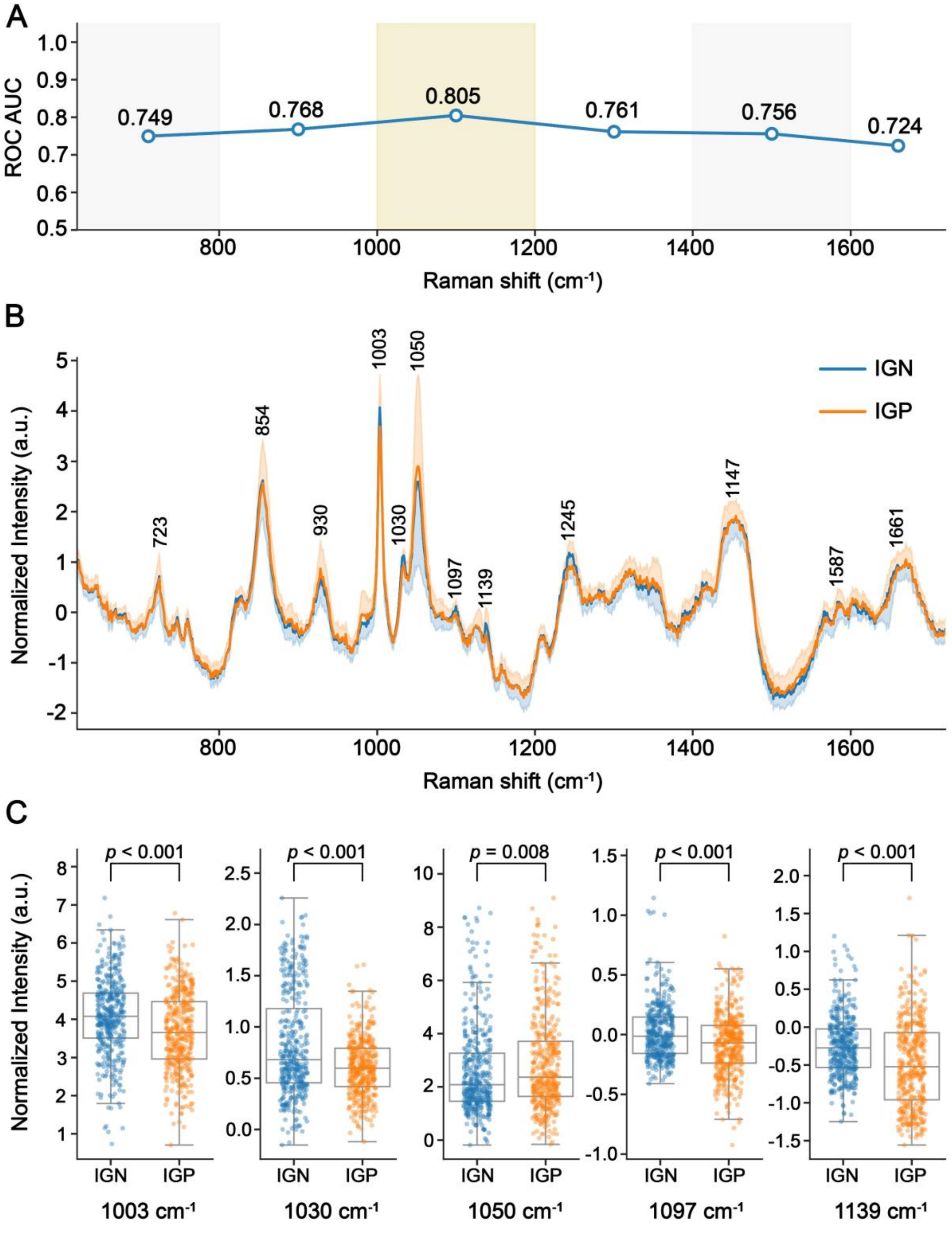

Fig D. Raman spectroscopic analysis of plasma samples for the discrimination between IGN and IGP groups. (A) Diagnostic performance across the fingerprint region. The line plot displays the Receiver Operating Characteristic Area Under the Curve (ROC AUC) values calculated for specific Raman shift sub-regions. The highest classification accuracy (AUC = 0.805) was achieved in the 1000–1200 cm^{-1} range (shaded in yellow), highlighting its

diagnostic significance. (B) Mean normalized Raman spectra. Comparative Raman profiles of the IGN (blue) and IGP (orange) groups within the 600–1750 cm^{-1} range. Shaded areas represent the standard deviation. Key biomolecular peaks are labeled, including Phenylalanine (1003 cm^{-1}), Proteins/C-C skeletal (1050 cm^{-1}) and Amide I (1661 cm^{-1}). (C) Statistical comparison of key Raman biomarkers. Box-and-whisker plots show the distribution of normalized intensities for five highly discriminative peaks (1003, 1030, 1050, 1097, and 1139 cm^{-1}). Individual data points represent independent samples. Statistical significance was determined using [Insert Statistical Test, e.g., Mann-Whitney U test], with p-values indicating highly significant differences ($p < 0.001$ to $p = 0.008$) between the two cohorts.

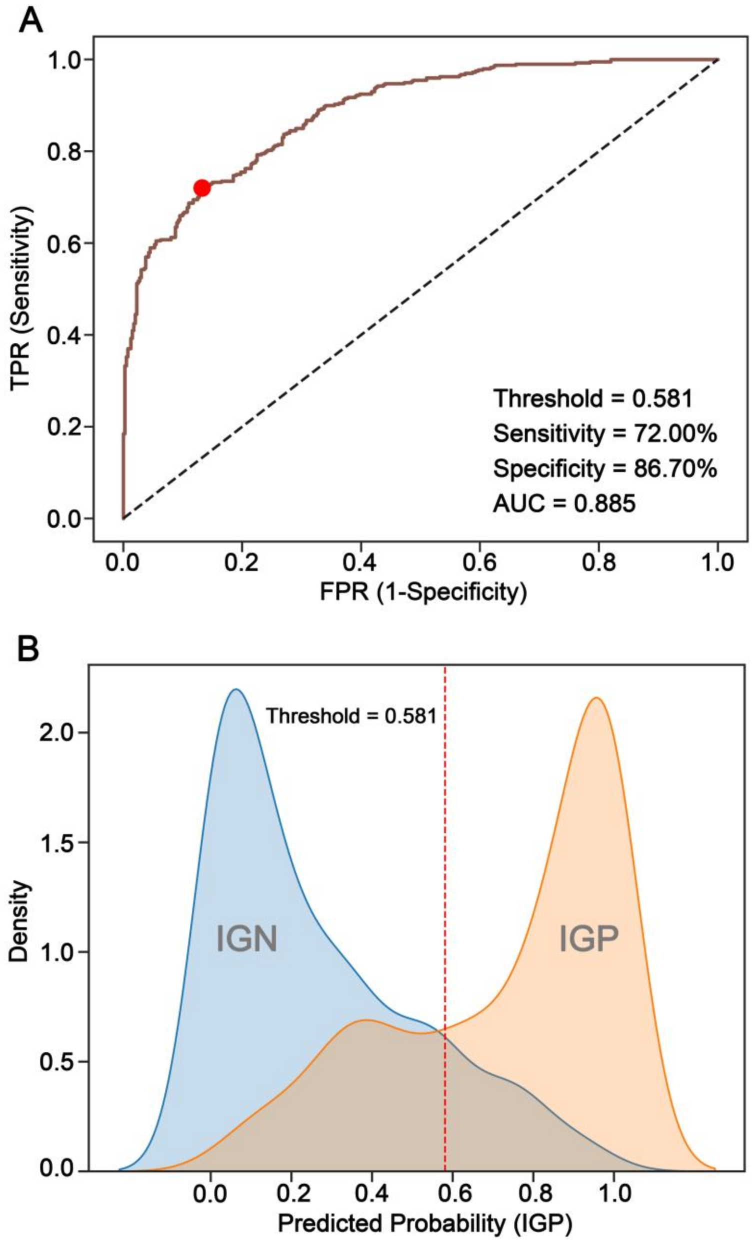

Fig E. Diagnostic performance and probability distribution of the 1D-CNN model. (A) Receiver Operating Characteristic (ROC) curve analysis for the training set using the optimized 1D-CNN architecture. The model achieved an Area Under the Curve (AUC) of 0.885. The optimal classification threshold was determined to be 0.581 using the Youden Index (indicated by the red dot), yielding a sensitivity of 72.00% and a specificity of 86.70%. (B) Density distribution of the predicted probabilities for the IGN (blue) and IGP (orange) groups. The vertical dashed red line represents the selected threshold (0.581), demonstrating the model's ability to effectively segregate the two cohorts, with the IGP group showing a clear shift towards higher predicted probability scores.

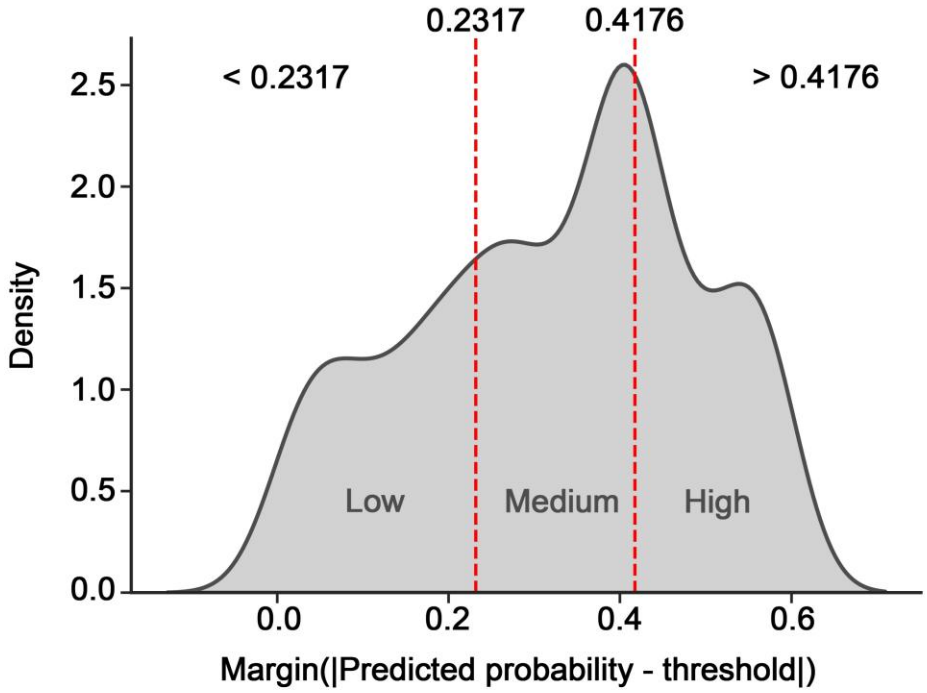

Fig F. Margin-based stratification of predictions. The distribution of prediction margins was quantified by calculating the absolute difference between the predicted probability and the optimal classification threshold (|predicted probability − threshold|). The density plot illustrates the stratification of predictions into three tiers (low, medium, and high) based on the 30th and 70th percentiles of the margin distribution. Vertical dashed red lines indicate the corresponding margin thresholds at 0.2317 and 0.4176. This stratification enables differentiation between predictions that are farther from the decision boundary and those closer to it, providing additional context for interpreting model outputs.

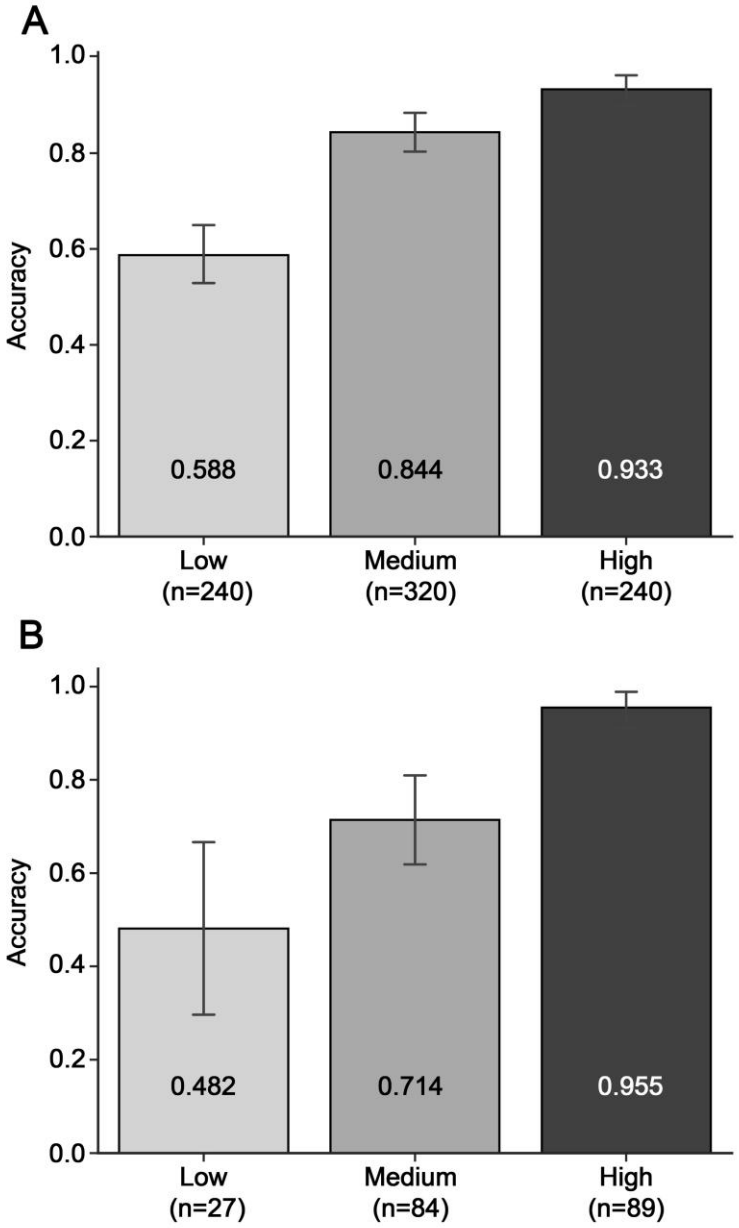

Fig G. Classification accuracy across margin-based prediction tiers. Classification accuracy was evaluated independently for each tier (low, medium, and high) for both the (A) training set and (B) validation set. Tiers were defined based on the margin between predicted probabilities and the classification threshold. The bar plots represent mean accuracy, with numerical values indicated within each bar. Error bars represent the [insert error bar type, e.g., 95% confidence intervals]. The sample size (n) for each tier is provided below the corresponding category labels. In both datasets, a positive relationship was observed between margin-based tiers and classification accuracy, with higher-margin groups showing improved performance (accuracy > 0.93 in the highest tier), supporting the use of margin-based stratification for interpreting model outputs.

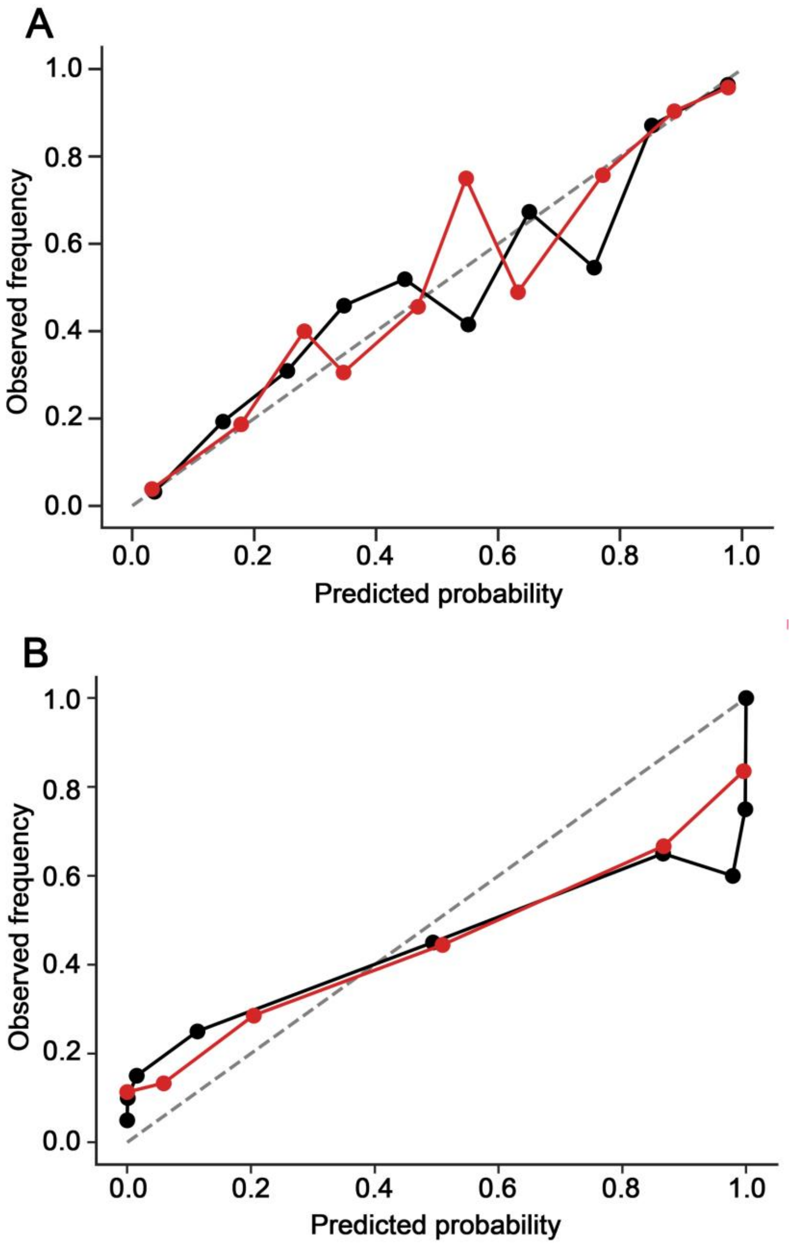

Fig H. Calibration plots of the diagnostic model before and after probability adjustment. Calibration curves were used to assess the agreement between predicted probabilities and observed outcomes for the (A) training set and (B) validation set. The dashed gray line represents the ideal "perfect calibration," where predicted probabilities match observed frequencies. The black lines indicate the model outputs before calibration, showing deviation from the ideal diagonal, while the red lines represent the calibrated outputs using isotonic regression. In both datasets, calibration shifted the predicted probabilities closer to the reference line, indicating improved agreement between predicted probabilities and observed outcomes. Calibration curves were generated using 10 bins (n = 10; quantile-based).

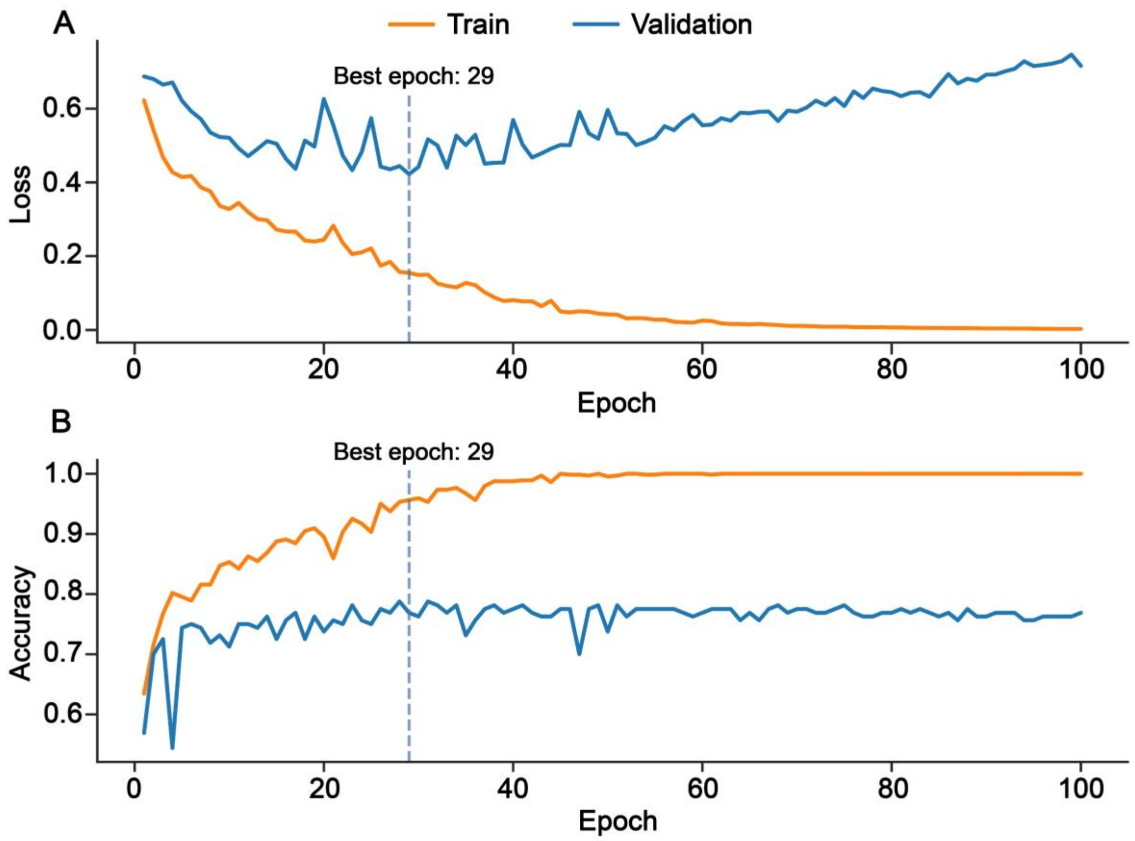

Fig I. Training and validation performance of the 1D-CNN model. The learning curves illustrate the optimization process over 100 epochs for (A) Loss and (B) Accuracy. The model was trained using 80% of the primary dataset, with a 20% internal validation split to monitor for overfitting. The orange lines represent the training set performance, while the blue lines represent the validation set performance. A vertical dashed line indicates the "Best Epoch" (Epoch 29), where the validation loss reached its minimum value. Beyond this point, the divergence between the training and validation loss suggests the onset of overfitting, justifying the selection of the model weights from epoch 29 for final deployment.

Supplementary Tables

Table A. Best hyperparameters and cross-validation performance of machine learning models obtained from 5-fold grid search on the training set.

Model	Best hyperparameters	AUC (mean ± SD)
LR	C = 0.01, penalty = L2, solver = saga	0.824 ± 0.021
LDA	shrinkage = 0.5, solver = lsqr	0.817 ± 0.025
SVM	C = 10, gamma = 0.001, kernel = RBF	0.860 ± 0.024
KNN	k = 7, metric = Manhattan, weights = distance	0.838 ± 0.025
RF	n_estimators = 200, max_depth = 20, min_samples_split = 2, min_samples_leaf = 4	0.865 ± 0.021
XGB	n_estimators = 200, max_depth = 6, learning_rate = 0.1	0.872 ± 0.025

Note: Best hyperparameters for each model were determined using grid search with 5-fold cross-validation on the training set. Model performance is reported as the mean ± standard deviation (SD) of the area under the receiver operating characteristic curve (AUC) across the cross-validation folds. LR = Logistic Regression; LDA = Linear Discriminant Analysis; SVM = Support Vector Machine; KNN = K-Nearest Neighbors; RF = Random Forest; XGB = Extreme Gradient Boosting.

Table B. ROC AUCs of the training set (5-fold CV, mean ± standard deviation) for models with varying numbers of convolutional layers, different numbers of kernels per layer, and mini-batch sizes. Models used a kernel size of [3,1] with average pooling (pooling size = [2,1]).

**Convolutional Layers**	**Number of kernels**	**Mini-batch size**			
		**16**	**32**	**64**	**128**
1	16	0.855 ± 0.020	0.855 ± 0.020	0.844 ± 0.012	0.835 ± 0.012
	32	0.848 ± 0.017	0.854 ± 0.020	0.849 ± 0.020	0.831 ± 0.011
	64	0.850 ± 0.012	0.852 ± 0.015	0.836 ± 0.020	0.808 ± 0.017
	128	0.773 ± 0.138	0.845 ± 0.020	0.837 ± 0.016	0.834 ± 0.015
2	16,16	0.866 ± 0.018	0.859 ± 0.015	0.819 ± 0.011	0.807 ± 0.029
	32,32	0.865 ± 0.016	0.858 ± 0.024	0.848 ± 0.015	0.825 ± 0.011
	64,64	0.866 ± 0.011	0.862 ± 0.017	0.826 ± 0.036	0.818 ± 0.032
	128,128	0.864 ± 0.018	0.862 ± 0.020	0.851 ± 0.014	0.823 ± 0.020
	16,32	0.863 ± 0.013	0.851 ± 0.019	0.842 ± 0.004	0.822 ± 0.012
3	16,16,16	0.865 ± 0.016	0.848 ± 0.015	0.808 ± 0.020	0.790 ± 0.016
	32, 32, 32	0.875 ± 0.022	0.844 ± 0.011	0.791 ± 0.058	0.806 ± 0.022
	64, 64, 64	0.877 ± 0.022	0.849 ± 0.021	0.831 ± 0.029	0.797 ± 0.020
	128, 128, 128	0.869 ± 0.024	0.860 ± 0.014	0.830 ± 0.005	0.808 ± 0.041
	16, 32, 64	0.873 ± 0.019	0.853 ± 0.012	0.819 ± 0.018	0.811 ± 0.036
4	16,16,16,16	0.854 ± 0.015	0.824 ± 0.010	0.768 ± 0.057	0.737 ± 0.039
	32, 32, 32, 32	0.867 ± 0.017	0.833 ± 0.011	0.769 ± 0.012	0.753 ± 0.019
	64, 64, 64, 64	0.873 ± 0.014	0.831 ± 0.017	0.789 ± 0.031	0.765 ± 0.037
	128, 128, 128, 128	0.875 ± 0.018	0.840 ± 0.015	0.805 ± 0.028	0.760 ± 0.025
	**16, 32, 64, 128**	**0.885 ± 0.022**	**0.854 ± 0.010**	**0.810 ± 0.028**	**0.774 ± 0.035**

Table C. ROC AUCs of the training set (5-fold CV, mean ± standard deviation) for models with varying numbers of kernels. All models used a kernel size of [3,1], a mini-batch size of 16, and average pooling (pooling size = [2,1]).

Convolutional Layers	Number of kernels	Kernel Size			
		[3,1]	[5,1]	[7,1]	[9,1]
1	16	0.855 ± 0.020	0.852 ± 0.013	0.856 ± 0.018	0.863 ± 0.017
2	16, 32	0.863 ± 0.013	0.869 ± 0.020	0.872 ± 0.021	0.870 ± 0.020
3	16, 32, 64	0.873 ± 0.019	0.875 ± 0.026	0.877 ± 0.026	0.870 ± 0.015
4	16, 32, 64, 128	**0.885 ± 0.022**	0.877 ± 0.026	0.871 ± 0.020	0.876 ± 0.018

Table D. ROC AUCs of the training set (5-fold CV, mean ± standard deviation) for models with varying pooling methods. All models used a kernel size of [3,1] and a mini-batch size of 16, with pooling size [2,1].

Convolutional Layers	Number of kernels	Average-pooling	Max-pooling
1	16	0.855 ± 0.020	0.853 ± 0.009
2	16, 32	0.863 ± 0.013	0.845 ± 0.013
3	16, 32, 64	0.873 ± 0.019	0.830 ± 0.028
4	16, 32, 64, 128	**0.885 ± 0.022**	0.831 ± 0.037

Table E. ROC AUCs of the training set (5-fold CV, mean ± standard deviation) for models with varying fully connected layers. All models used a kernel size of [3,1], a mini-batch size of 16, and average pooling (pooling size = [2,1]).

**Convolutional Layers**	**Number of kernels**	**Size of fully connected layer**			
		**32**	**64**	**128**	**256**
1	16	0.847 ± 0.018	0.853 ± 0.018	0.855 ± 0.020	0.856 ± 0.025
2	16, 32	0.853 ± 0.014	0.864 ± 0.014	0.863 ± 0.013	0.860 ± 0.018
3	16, 32, 64	0.869 ± 0.011	0.869 ± 0.013	0.873 ± 0.019	0.871 ± 0.015
4	16, 32, 64, 128	0.862 ± 0.017	0.874 ± 0.020	**0.885 ± 0.022**	0.877 ± 0.024

Table F. ROC AUCs of the training set (5-fold CV, mean ± standard deviation) for models with varying learning rates. All models used a kernel size of [3,1], a mini-batch size of 16, a fully connected layer with 128 units, and average pooling (pooling size = [2,1]).

**Convolutional Layers**	**Number of kernels**	**Size of learning rate**		
		**0.0001**	**0.0003**	**0.0005**
1	16	0.855 ± 0.020	0.857 ± 0.012	0.847 ± 0.016
2	16, 32	0.863 ± 0.013	0.864 ± 0.012	0.848 ± 0.022
3	16, 32, 64	0.873 ± 0.019	0.873 ± 0.016	0.866 ± 0.016
4	16, 32, 64, 128	**0.885 ± 0.022**	0.866 ± 0.017	0.856 ± 0.024

Table G. Raman shifts positions and corresponding vibrational mode assignments for key biochemical constituents identified in human plasma.

Raman Shift (cm^{-1})	Assignments	Reference(s)
723	C-N symmetric stretching	[1]
854	Polysaccharides / C-O-C skeletal mode	[2]
930	Proteins (Proline, Valine) / C-C stretching	[2]
1003	Phenylalanine / C-C ring breathing	[2, 3]
1030	Proteins (Collagen, Keratin) / C-C skeletal	[2, 4]
1050	C-N and C-C stretching	[3, 4]
1097	Nucleic acids / Phosphate stretching	[2]
1139	Proteins CH_2 and CH_3 bending	[5, 6]
1245	Proteins (Amide III)	[2]
1447	Proteins and Lipids CH_2 bending	[1]
1587	Phenylalanine and Hydroxyproline	[2]
1661	Proteins (Amide I) / C=O stretching	[3]

Note: Assignments are derived from literature comparisons. Abbreviations: Amide I/III, protein secondary structure bands; C–C, carbon–carbon bond; C–N, carbon–nitrogen bond; C=O, carbon–oxygen double bond; CH_2, methylene; CH_3, methyl. All assignments represent specific vibrational modes (stretching, bending, or ring breathing) associated with the respective biomolecular markers.

Table H Analysis of processing time for plasma samples with complete participant information.

Number of Iteration	Time of Analysis (seconds)			
	**Blinded #1**	**Blinded #2**	**Blinded #3**	**Blinded #4**
1	10.61	8.63	10.43	7.52
2	9.41	7.81	7.63	6.62
3	9.37	10.49	7.26	7.86
4	8.83	8.09	7.70	7.55
5	8.75	8.99	9.34	8.62
6	9.26	9.18	8.22	8.39
7	9.15	8.79	10.01	9.04
8	9.47	9.51	8.77	10.22
9	10.57	10.29	6.76	7.74
10	8.79	10.29	8.79	8.35
**Average Analysis Time (10 replicates, 4 samples) = 8.83 seconds**				

Note: Total analysis time (in seconds) includes both spectral analysis and final report generation for each sample across 10 replicates. Average Analysis Time (across 10 replicates and 4 samples): 8.83 seconds. Each sample was analyzed in 10 replicates using a laptop equipped with an Intel Core i5-10300H (2.50 GHz), 16.0 GB RAM, and an NVIDIA GeForce GTX 1650.

Table I. Detailed prediction results of the TB-SERS Analyzer for the blinded external test set (n = 20).

Input file	Prediction Probability (%)		Final interpretation	Prediction Tier
	Negative	Positive		
Blinded #1 on SERS.txt	99.98%	0.02%	IGRA-negative	High
Blinded #2 on SERS.txt	63.22%	36.78%	IGRA-negative	Low
Blinded #3 on SERS.txt	86.09%	13.91%	IGRA-negative	Medium
Blinded #4 on SERS.txt	51.14%	48.86%	IGRA-negative	Low
Blinded #5 on SERS.txt	94.72%	5.28%	IGRA-negative	Medium
Blinded #6 on SERS.txt	98.63%	1.37%	IGRA-negative	Medium
Blinded #7 on SERS.txt	98.81%	1.19%	IGRA-negative	Medium
Blinded #8 on SERS.txt	100.00%	0.00%	IGRA-negative	High
Blinded #9 on SERS.txt	100.00%	0.00%	IGRA-negative	High
Blinded #10 on SERS.txt	92.23%	7.77%	IGRA-negative	Medium
Blinded #11 on SERS.txt	99.99%	0.01%	IGRA-negative	High
Blinded #12 on SERS.txt	92.29%	7.71%	IGRA-negative	Medium
Blinded #13 on SERS.txt	0.01%	99.99%	IGRA-positive	High
Blinded #14 on SERS.txt	2.67%	97.33%	IGRA-positive	Medium
Blinded #15 on SERS.txt	11.16%	88.84%	IGRA-positive	Medium
Blinded #16 on SERS.txt	33.36%	66.64%	IGRA-positive	Low
Blinded #17 on SERS.txt	9.65%	90.35%	IGRA-positive	Medium
Blinded #18 on SERS.txt	3.54%	96.46%	IGRA-positive	Medium
Blinded #19 on SERS.txt	0.01%	99.99%	IGRA-positive	High
Blinded #20 on SERS.txt	0.05%	99.95%	IGRA-positive	High

Note: Probabilities were generated using the 1D-CNN model by the TB-SERS Analyzer. Prediction Margin is defined as the absolute difference between the predicted probability and the optimal classification threshold. Margin-based predictions are categorized into three tiers on the prediction margin: High (> 0.4176), Medium (0. 0.2317–0.4176), and Low (< 0.2317).

Supplementary References

1. Masui K, Nawa Y, Tokumitsu S, Nagano T, Kawarai M, Tanaka H, et al. Detection of Glutamate Encapsulated in Liposomes by Optical Trapping Raman Spectroscopy. **ACS Omega**. 2022;7.
2. Movasaghi Z, Rehman S, Rehman I. Raman Spectroscopy of Biological Tissues. **Applied Spectroscopy Reviews**. 2007;42:493-541.
3. Lin D, Pan J, Huang H, Chen G, Qiu S, Shi H, et al. Label-free blood plasma test based on surface-enhanced Raman scattering for tumor stages detection in nasopharyngeal cancer. **Scientific Reports**. 2014;4(1):4751.
4. Zhu J, Zhou J, Guo J, Cai W, Liu B, Wang Z, et al. Surface-enhanced Raman spectroscopy investigation on human breast cancer cells. **Chem Cent J**. 2013;7(1):37.
5. Risi R, Manti L, Perna G, Lasalvia M, Capozzi V, Delfino I, et al. X-ray radiation-induced effects in human mammary epithelial cells investigated by Raman microspectroscopy2012. 50 p.
6. Zhang Q, Deng M, Gao Q, Zhou X, Guo Y, Wang Y, et al. Performance of Raman Spectroscopy in biopsy tissue for rapid diagnosis of Tracheobronchial Tuberculosis: A prospective study. **Photodiagnosis and Photodynamic Therapy**. 2025;53:104556.
