# Supplementary material for: TB-SERS analyzer: Analysis tool for tuberculosis prediction based on Raman spectroscopy with machine learning and convolutional neural network
Source: PLoS Comput Biol. 2026 Jul 14;22(7):e1014397. doi: 10.1371/journal.pcbi.1014397 (PMC13379083; doi:10.1371/journal.pcbi.1014397)
Supplement: Attachment — Submitted filename: PCOMPBIOL-D-25-02316_review.pdf [file pcbi.1014397.s002.pdf]

Paper title: TB-SERS Analyzer: Analysis tool for tuberculosis prediction based on Raman spectroscopy with machine learning and convolutional neural network

Summary:
This manuscript proposes a new framework, TB-SERS Analyzer, a Python-based software with a GUI to analyze SERS data for TB prediction. The study follows a very straightforward methodology. It performs data preparation, uses simple ML models and a 1D-CNN for TB prediction and generates reports in pdf format.

Major comments:

- The introduction is missing a state-of-the-art review about ML/DL approaches for Tb prediction (for SERS and others). A quick search:
  https://www.sciencedirect.com/science/article/pii/S2001037022004366
  https://arxiv.org/pdf/2306.05653
- Cannot use the tool (Ubuntu 24.04.3 LTS). When doing "python TB-SERS-Analyzer.py" it raises the following error: "ImportError: cannot import name 'FontFace' from 'fpdf.fonts'" - It worked after running: "pip install fpdf2"
- After fixing the previous issue, I still got the following error after trying to generate a report: "File "<__array_function__ internals>", line 200, in concatenate
  ValueError: all the input array dimensions except for the concatenation axis must match exactly, but along dimension 0, the array at index 0 has size 1011 and the array at index 1 has size 151884
  Aborted (core dumped)"
- Why not use CV for model selection instead of different train-test proportions?
- Data, models and training scripts are not publicly available?

Minor comments:

- Line 25: "In the context of tuberculosis (TB) diagnosis, RS/SERS offers significant potential." - in what? Complete this!
- Acronyms must be written in full at first appearance and then in the abbreviated form (e.g., IGRA, TP, TN, FP, FN) - REVER ISTO - Só se verificou no abstract
- Line 33-34: The term fine-tuned in ML refers to another procedure (), here the authors mean trained.
- CHECK THIS: 1000 samples - split 60/40 - should they use CV instead?
- Lines 205-206: "The uploaded file is read using the NumPy package [18], depending on the file type." What are the possible file types?
- Line 209: in "tolerance of 3" add units.
- Lines 210-212: "The software issues a warning if the Spearman correlation (ρ) between a blinded sample and the reference model falls below the threshold." What are the implications of this?

Others:

- Regarding the "Validation of the TB-SERS Analyzer with blinded plasma samples" section: 4 samples for model validation is insufficient to draw reliable conclusions…
