# Supplementary material for: TB-SERS analyzer: Analysis tool for tuberculosis prediction based on Raman spectroscopy with machine learning and convolutional neural network
Source: PLoS Comput Biol. 2026 Jul 14;22(7):e1014397. doi: 10.1371/journal.pcbi.1014397 (PMC13379083; doi:10.1371/journal.pcbi.1014397)
Supplement: Attachment — Submitted filename: Response to Reviewers.docx [file pcbi.1014397.s004.docx]

**Faculty of Medicine**

Khon Kaen University

123 Mittraparb Road

Nai-Meung, Meung District

Khon Kaen, Thailand 40002

Tel/Fax: (66) 43 363 808

<https://www.kku.ac.th/?l=en>

kiatichai@kku.ac.th	April 16, 2026

**Dear Prof. Feilim Mac Gabhann**

**Editors-in-Chief, and Editorial Team, *PLOS Computational Biology***

We sincerely thank the editors and reviewers for their valuable comments and constructive suggestions. We have carefully revised the manuscript in response to all concerns, and each point is addressed in detail in the accompanying point-by-point response.

In summary, the manuscript has been substantially improved in several important ways. In response to **Reviewer 1**, we expanded the Introduction to include a state-of-the-art review of machine learning and deep learning approaches for TB prediction, updated the software dependencies and documentation to resolve runtime issues, clarified supported input file formats and preprocessing steps, and revised the evaluation strategy to use cross-validation–based model selection rather than relying on multiple train–test splits. We also improved the availability of data and code resources to strengthen reproducibility.

In response to **Reviewer 2**, we added a brief characterization of the input SERS spectra, provided a much more detailed and standardized description of the preprocessing workflow, and included a new biological-spectral feature analysis to improve interpretability and highlight the spectral regions most relevant for distinguishing LTBI from non-TB cases.

In response to **Reviewer 3**, we strengthened the methodological rigor of the study by expanding the external blind validation cohort, revising the model development workflow to eliminate potential data leakage, clarifying the limitations of IGRA-derived labels, replacing the original confidence estimation approach with a more interpretable margin-based prediction analysis, and adding discussion of clinical relevance, software platform limitations, and computational requirements.

We believe these revisions have substantially improved the clarity, robustness, reproducibility, and interpretability of the manuscript. We hope the revised version is now suitable for publication in *PLOS Computational Biology*. The resubmission of this manuscript has been approved by all authors.

Sincerely,

**Professor Kiatichai Faksri**
Department of Microbiology, Faculty of Medicine

Khon Kaen University, Thailand

On behalf of all authors

**Point-by-point response to reviewer's**

**Reviewer 1**

**Major comments:**

1. The introduction is missing a state-of-the-art review about ML/DL approaches for Tb

prediction (for SERS and others). A quick search:

https://www.sciencedirect.com/science/article/pii/S2001037022004366

https://arxiv.org/pdf/2306.05653

**Response:** Thank you for this valuable comment. We agree that the original introduction did not sufficiently address recent state-of-the-art ML/DL approaches for TB prediction. In response, we have revised the Introduction to include an additional paragraph summarizing recent advances in ML and DL for TB classification, including both SERS/Raman-based studies and other TB prediction modalities such as chest X-ray imaging, clinical metadata, and microbiological data with relevant citations (lines 75–94).

2. Cannot use the tool (Ubuntu 24.04.3 LTS). When doing “python

TB-SERS-Analyzer.py” it raises the following error: “ImportError: cannot import name

'FontFace' from 'fpdf.fonts'” - It worked after running: “pip install fpdf2”

**Response:** We have resolved this issue by replacing the older fpdf library with fpdf2, which supports the FontFace class. The installation instructions have been updated accordingly, and this change has been reflected in the manuscript (Section Availability and future directions, line 483).

3. After fixing the previous issue, I still got the following error after trying to generate a

report: “File "<__array_function__ internals>", line 200, in concatenate

ValueError: all the input array dimensions except for the concatenation axis must

match exactly, but along dimension 0, the array at index 0 has size 1011 and the

array at index 1 has size 151884

Aborted (core dumped)”

**Response:** The runtime error during report generation was caused by inconsistent input data shapes. To address this, we have ensured proper handling of input data. The blind dataset used for testing the software (20 blind samples) has been deposited in Zenodo and is accessible at <https://doi.org/10.5281/zenodo.17535166> (Section Data availability statement, lines 558-559).

4. Why not use CV for model selection instead of different train-test proportions?

**Response:** We agree that cross-validation (CV) provides a more robust framework for model selection. Therefore, we revised the analysis to incorporate a CV-based model selection strategy instead of relying on multiple train–test splits.

Specifically, the dataset (n=1,000) was first partitioned into training (n=800) and validation (n=200) sets (**Fig 1**). Within the training set, a 5-fold stratified cross-validation was implemented. This CV scheme was used for hyperparameter optimization via GridSearchCV for six conventional machine learning models, as well as for architecture tuning of the 1D-CNN.

Importantly, out-of-fold predictions generated during CV were used to provide an unbiased estimate of model performance. Model selection was then based on the mean ROC-AUC across the CV folds.

These revisions have been made in:

- Methods (Model Optimization, lines 150–170)

- Results (Comparative performance of ML algorithms and 1D-CNN, lines 327–342)

5. Data, models and training scripts are not publicly available?

**Response:** To address this, we have improved the availability of data and code to enhance reproducibility. Specifically, the shareable components of the preprocessing and analysis workflow are provided (<https://github.com/jkeisiri/TB-SERS-Analyzer/tree/main/analysis>).

In addition, to facilitate independent evaluation and software testing, the blind dataset (n = 20) has been deposited in Zenodo ([https://doi.org/[10.5281/zenodo.17535165](https://doi.org/10.5281/zenodo.17535165)](https://doi.org/10.5281/zenodo.17535166)) and is publicly accessible (lines 558–561).

**Minor comments:**

6. Line 25: “In the context of tuberculosis (TB) diagnosis, RS/SERS offers significant

potential.” - in what? Complete this!

**Response:** We have completed the sentence to clarify the context (line 26).

7. Acronyms must be written in full at first appearance and then in the abbreviated form

(e.g., IGRA, TP, TN, FP, FN) - REVER ISTO - Só se verificou no abstract

**Response:** We have revised the manuscript to ensure that all acronyms are written in full at their first appearance, followed by the corresponding abbreviations throughout the text.

This revision has been applied consistently across the entire manuscript.

8. Line 33-34: The term fine-tuned in ML refers to another procedure (), here the

authors mean trained.

**Response:** We have replaced “fine-tuned” with “trained and optimized via five-fold stratified cross-validation” (lines 34–35).

9. CHECK THIS: 1000 samples - split 60/40 - should they use CV instead?

**Response:** The dataset (n=1,000) was partitioned into a training set (n=800) and an independent validation set (n=200). All model development, including hyperparameter tuning via five-fold stratified cross-validation and 1D-CNN architecture optimization, was performed exclusively on the training set.

The validation set was strictly held out and used only for final performance evaluation, ensuring an unbiased assessment of model generalization. In addition, model performance was further confirmed using an external test set (n=20).

These revisions have been made in:

- Methods (Performance evaluation and final classification algorithms, lines 208–225).

10. Lines 205-206: “The uploaded file is read using the NumPy package [18], depending

on the file type.” What are the possible file types?

**Response:** We have clarified the supported input format. The software currently supports text-based (.txt) files only, as no other file formats were available for evaluation (lines 287–288).

11. Line 209: in “tolerance of 3” add units.

**Response:** We have added the units for “tolerance of 3,” which is now specified as 3 cm^-1^ (lines 291).

12. Lines 210-212: “The software issues a warning if the Spearman correlation (ρ)

between a blinded sample and the reference model falls below the threshold.” What

are the implications of this?

**Response:** We have clarified the implications of this warning. The spectral similarity of Raman intensity profiles is assessed using Spearman correlation (*ρ*). A warning is issued when ρ falls below a predefined threshold, indicating potentially poor spectral quality or that the sample lies outside the training data distribution, which may compromise the reliability of the prediction (lines 291–298).

Others:

13. Regarding the “Validation of the TB-SERS Analyzer with blinded plasma samples”

section: 4 samples for model validation is insufficient to draw reliable conclusions...

**Response:** We acknowledge that a small number of blind samples alone may be insufficient to draw definitive conclusions. To address this, model development and primary evaluation were based on a larger dataset (n=1,000), which was partitioned into a training set (n=800) and an independent validation set (n=200).

The blinded samples (n=20) were used as an additional external test set to further assess model generalizability. Consistent performance across the internal validation set and the external test set supports the reliability of the model predictions (**Table 2**).

This revision has been made in:

- Results (Validation and external blind testing of the TB-SERS Analyzer, lines 418–445).

_______________________________________________________________________

**Reviewer 2**

1. Although the origin of samples and their compounds subjected to the SERS study can be understood by reading Ref. 13, it would be better to characterize them shortly at the beginning of the manuscript; a potential user should clearly know the spectra of what should be uploaded into the program.

**Response:** To address this, we have added a brief characterization of the samples at the beginning of the manuscript to clarify the type of spectra expected for input into the program. Additional details on their spectral features are provided in **Fig A in S1 Appendix** (Section Data preparation and preprocessing, lines 119–141).

2. The preprocessing procedure requires further clarification in more detail: since the ML-based algorithms, which take into account not only peak positions but also their intensities, the meaning of relative arbitrary units must be clarified: Was the norming to the global maximum after the preliminary filtering and the baseline extraction applied? Or to some reference peak? Was a processed spectrum obtained shifted down to correspond to its minimal point to zero before normalization, etc? This information is crucial because Raman spectra obtained from biological samples are, as a rule, ‘dirty’; it is not a clear sequence of well-expressed peaks typical for chemistry. Whence, the preprocessing of such spectra may significantly affect the result. In particular, all spectra used for training and tests in this work were obtained with the same equipment and preprocessing procedure. But what happens when one will use a spectrum obtained in another lab? The standardization of input data must be uniform and well-described in detail.

**Response:** To address this, we have clarified and standardized the preprocessing procedure in detail to ensure reproducibility and consistency across samples. The updated pipeline includes: (i) removal of cosmic ray spikes using a modified Z-score method, (ii) outlier detection via PCA with robust Mahalanobis distance filtering, (iii) baseline correction using a third-order polynomial, (iv) shifting each spectrum so that its minimum intensity is set to zero, (v) normalization using standard normal variate (SNV), and (vi) averaging spectra from each sample to obtain a representative spectrum.

All spectra used for model development and evaluation were acquired using the same instrument and processed using this consistent pipeline. In addition, the preprocessing workflow has been made available at <https://github.com/jkeisiri/TB-SERS-Analyzer/tree/main/analysis> to support reproducibility. These steps ensure standardized input is suitable for machine learning analysis, particularly for biologically derived Raman spectra.

These revisions have been made in:

- Methods (Data preparation and preprocessing, lines 125–141).

3. Although the performance of the proposed software is well discussed from the computational point of view, it is still a ‘black box’ like most of the NN algorithms. But some additional biologico-spectral analysis of spectral features in charge of the response seems possible and will strengthen the work. In particular, it is interesting to discuss specific spectral regions that are most valuable for distinguishing between LTBI and non-TB cases (an example of such an analysis applied to another SERS-related problem can be found, e.g., in the work https://doi.org/10.7717/peerj.18830.) This can be done easily, either simply subdividing the whole spectrum into parts and processing shorter wavelength regions, or multiplying the spectrum by finite-length filters with different centering.

**Response:** We thank the reviewer for this valuable suggestion to improve the biological interpretability of our models. To address this, we have incorporated additional biological-spectral analysis. A new section, biological significance and spectral feature analysis, has been added. The fingerprint region (620–1720 cm⁻¹) was partitioned into six sub-regions (620–800, 800–1000, 1000–1200, 1200–1400, 1400–1600, and 1600–1720 cm^-1^), and the diagnostic performance (ROC-AUC) of each sub-region was independently evaluated using the best-performing model under five-fold cross-validation on the training set.

Prominent Raman peaks within high-performing regions were further analyzed, and their associations with key biomolecular components (e.g., lipids, proteins, and nucleic acids) were statistically evaluated using the Mann–Whitney U test to compare intensity distributions between groups.

These additions have been made in:

- Methods (Biological significance and spectral feature analysis, lines 236–250).

- Results (Sub-region performance analysis and biomolecular interpretation, lines 350–370).

___________________________________________________________________________

**Reviewer 3**

**Major Concerns:**

1. The most significant weakness is the blind validation cohort of only four samples. While the concordance looks impressive in Table 2, four samples is wholly insufficient to draw meaningful conclusions about real-world diagnostic performance. A tool being proposed for clinical TB screening should be validated on a substantially larger independent cohort, ideally from a different collection site or patient population.

**Response:** We thank the reviewer for this important comment. We acknowledge that a very small blind validation cohort is insufficient to draw definitive conclusions regarding real-world performance. To address this, we have expanded the external blind test set from 4 to 20 samples and clarified its role as an independent evaluation dataset.

Model development and primary evaluation were conducted using a larger dataset (n=1,000), with a strict separation between training (n=800) and independent validation (n=200) sets. The validation set was not used during model training or optimization, ensuring an unbiased assessment of performance.

Consistent results observed across the internal validation set and the expanded external test set support the reliability and generalizability of the proposed approach (**Table 2).**

This revision has been made in:

- Methods (Performance evaluation and final classification algorithms, lines 208–225).

- Results (Validation and external blind testing of the TB-SERS Analyzer, lines 418–445).

2. The retraining procedure raises a data leakage concern. The authors retrain the final 1D-CNN on the full 1,000-sample dataset using 80% training and 20% validation, but these are the same samples used for model selection and threshold tuning via LOOCV. Without a truly held-out external test set, reported performance metrics are likely optimistic.

**Response:** We thank the reviewer for highlighting this important concern regarding potential data leakage. To address this, we have revised the model optimization workflow (**Fig 1**) to ensure a strict separation between training, validation, and external testing.

Specifically, the final 1D-CNN was trained exclusively on the training set (n=800). The internal validation set (n=200) was not used during model training, hyperparameter optimization, or threshold tuning, and was reserved solely for independent performance evaluation. In addition, an external blind test set (n=20) was used to further assess model generalizability.

These changes eliminate the risk of data leakage and ensure that the reported performance metrics reflect unbiased model evaluation (**Table 2**).

This revision has been made in:

- Methods (Fig 1, lines 172–184).

- Results (Table 2, line 470).

3. The choice of IGRA as the reference standard is acknowledged as imperfect, but its implications deserve more discussion. IGRA cannot distinguish active TB from LTBI, has variable sensitivity in immunocompromised populations, and performs poorly in high-burden low-income settings, precisely where this tool is presumably intended to be deployed. The authors should discuss how IGRA-derived labels may have introduced systematic misclassification into the training data.

**Response:** We thank the reviewer for this important comment. To address this, we have clarified this limitation in the manuscript and emphasized that model predictions should be interpreted in the context of IGRA-defined immune response status rather than definitive clinical TB diagnosis. We have also highlighted this as a consideration for future development and validation.

This revision has been made in:

- Section (Availability and future directions, lines 491–499).

4. The confidence interval methodology is unconventional. Using the 33rd and 66th percentiles of predicted probabilities across TP/TN/FP/FN groups to define confidence tiers is not a standard approach and is not well-justified. The authors should either provide a statistical rationale or adopt a more established uncertainty quantification method.

**Response:** We thank the reviewer for this important comment regarding the confidence estimation approach. To address this, we have replaced the original percentile-based method with a margin-based prediction analysis, which provides a more interpretable and statistically grounded measure of prediction certainty.

In this revised approach, the absolute margin between each predicted probability and the optimal classification threshold (|predicted probability − threshold|) is calculated using out-of-fold predictions from five-fold stratified cross-validation, ensuring unbiased estimation. These margin values were used to stratify predictions into three tiers (low, medium, and high) based on the 30th and 70th percentiles, reflecting the distance from the decision boundary.

To further support this approach, classification performance was evaluated within each tier, and calibration analysis using isotonic regression was performed to assess agreement between predicted probabilities and observed outcomes.

This revision has been made in:

- Methods (Threshold determination and margin-based prediction analysis, lines 252–

279).

- Results (Threshold optimization and performance across margin-based prediction tiers in TB-SERS Analyzer, lines 372–399).

**Minor Concerns:**

5. The 79.75% accuracy at the 60:40 split, while the best-performing configuration, remains modest for a clinical screening tool. Some clinical context on what sensitivity/specificity trade-offs would be acceptable in TB screening practice would help readers interpret whether this level of performance is clinically useful.

**Response:** We thank the reviewer for this important comment. To address this, we have revised the manuscript to report model performance in terms of both sensitivity and specificity, providing a more clinically relevant assessment of screening performance (lines 36–38).

In addition, we have clarified the interpretation of these metrics in the context of TB screening, where sensitivity is prioritized to minimize missed cases, while maintaining reasonable specificity. Performance is reported for both the internal validation set and the external blind test set to reflect real-world applicability (**Table 2**).

6. The software is compiled only for Windows, despite the claim of platform independence. This is a practical barrier for various settings and should either be resolved or more clearly caveated.

**Response:** We thank the reviewer for pointing out this limitation. We acknowledge that providing the software only as a precompiled Windows executable may limit usability in non-Windows environments.

To address this, we have clarified that while the TB-SERS Analyzer source code is platform-independent and can be compiled on other operating systems, official precompiled versions are currently available only for Windows. This limitation and its implications for users are now explicitly stated in the manuscript (lines 105–108).

This revision has been made in:

- Section (Availability and future directions, lines 478–482).

7. The manuscript would benefit from a brief discussion of computational requirements, as the tool is positioned for resource-limited settings, yet no mention is made of minimum hardware specifications.

**Response:** We thank the reviewer for this helpful suggestion. To address this, we have added a brief description of the computational requirements of the TB-SERS Analyzer.

The software was developed and tested on a system equipped with an Intel® Core™ i5-10300H CPU, 16 GB RAM, and an NVIDIA GeForce GTX 1650 GPU. To support use in resource-limited settings, we indicate that the tool can run on systems with a multi-core processor and at least 4 GB of RAM. The implementation relies on lightweight libraries, enabling efficient execution without high computational demands.

We also note that performance has not been systematically evaluated across diverse hardware configurations.

This revision has been made in:

- Section (Software architecture and implementation, lines 109–115).
