# Supplementary material for: TB-SERS analyzer: Analysis tool for tuberculosis prediction based on Raman spectroscopy with machine learning and convolutional neural network
Source: PLoS Comput Biol. 2026 Jul 14;22(7):e1014397. doi: 10.1371/journal.pcbi.1014397 (PMC13379083; doi:10.1371/journal.pcbi.1014397)
Supplement: Peer review history

# Peer review: Decision Letter 0

**DOI:** 10.1371/journal.pcbi.1014397.r001

**Authors:** Dominik Wodarz, Miguel Francisco de Rocha

3 Mar 2026

-->PCOMPBIOL-D-25-02316

TB-SERS Analyzer: Analysis tool for tuberculosis prediction based on Raman spectroscopy with machine learning and convolutional neural network

PLOS Computational Biology

Dear Dr. Faksri,

Thank you for submitting your manuscript to PLOS Computational Biology. After careful consideration, we feel that it has merit but does not fully meet PLOS Computational Biology's publication criteria as it currently stands. Therefore, we invite you to submit a revised version of the manuscript that addresses the points raised during the review process.

Please submit your revised manuscript by May 03 2026 11:59PM. If you will need more time than this to complete your revisions, please reply to this message or contact the journal office at ploscompbiol@plos.org. When you're ready to submit your revision, log on to https://www.editorialmanager.com/pcompbiol/ and select the 'Submissions Needing Revision' folder to locate your manuscript file.

Please include the following items when submitting your revised manuscript:

* A letter that responds to each point raised by the editor and reviewer(s). You should upload this letter as a separate file labeled 'Response to Reviewers'. This file does not need to include responses to formatting updates and technical items listed in the 'Journal Requirements' section below.

* A marked-up copy of your manuscript that highlights changes made to the original version. You should upload this as a separate file labeled 'Revised Manuscript with Track Changes'.

* An unmarked version of your revised paper without tracked changes. You should upload this as a separate file labeled 'Manuscript'.

If you would like to make changes to your financial disclosure, competing interests statement, or data availability statement, please make these updates within the submission form at the time of resubmission. Guidelines for resubmitting your figure files are available below the reviewer comments at the end of this letter

We look forward to receiving your revised manuscript.

Kind regards,

Miguel Francisco de Almeida Pereira de Rocha

Academic Editor

PLOS Computational Biology

Dominik Wodarz

Section Editor

PLOS Computational Biology

**Journal Requirements:**

1) Please upload all main figures as separate Figure files in .tif or .eps format. For more information about how to convert and format your figure files please see our guidelines:

https://journals.plos.org/ploscompbiol/s/figures

2) Some material included in your submission may be copyrighted. According to PLOSu2019s copyright policy, authors who use figures or other material (e.g., graphics, clipart, maps) from another author or copyright holder must demonstrate or obtain permission to publish this material under the Creative Commons Attribution 4.0 International (CC BY 4.0) License used by PLOS journals. Please closely review the details of PLOSu2019s copyright requirements here: PLOS Licenses and Copyright. If you need to request permissions from a copyright holder, you may use PLOS's Copyright Content Permission form.

Please respond directly to this email and provide any known details concerning your material's license terms and permissions required for reuse, even if you have not yet obtained copyright permissions or are unsure of your material's copyright compatibility. Once you have responded and addressed all other outstanding technical requirements, you may resubmit your manuscript within Editorial Manager.

Potential Copyright Issues:

i) Figures 1, 2, Supplementary figures (A, L, and O). Please confirm whether you drew the images / clip-art within the figure panels by hand. If you did not draw the images, please provide (a) a link to the source of the images or icons and their license / terms of use; or (b) written permission from the copyright holder to publish the images or icons under our CC BY 4.0 license. Alternatively, you may replace the images with open source alternatives. See these open source resources you may use to replace images / clip-art:

- https://commons.wikimedia.org

- https://openclipart.org/.

ii) The following Figures contain a logo or branding: 2, and Supplementary figure (O). We are not permitted to publish this under our CC-BY 4.0 license, even with permission. We ask that you please remove or replace it.

iii) The following Figures contain screenshots: 2, and Supplementary figure (P). We are not permitted to publish these under our CC-BY 4.0 license, websites are usually intellectual property and are copyrighted.This includes peripheral graphics of the web browser such as icons and button. We ask that you please remove or replace it.

3) Thank you for stating that "The newly generated Raman spectral data (four blind samples) have been deposited in Zenodo and are accessible at https://doi.org/10.5281/zenodo.17535166." We noted that the data files are restricted to users with access. Please note that, though access restrictions are acceptable now, your entire minimal dataset will need to be made freely accessible if your manuscript is accepted for publication. This policy applies to all data except where public deposition would breach compliance with the protocol approved by your research ethics board.

4) Please amend your detailed Financial Disclosure statement. This is published with the article. It must therefore be completed in full sentences and contain the exact wording you wish to be published.

1) State the initials, alongside each funding source, of each author to receive each grant. For example: "This work was supported by the National Institutes of Health (####### to AM; ###### to CJ) and the National Science Foundation (###### to AM)."

2) State what role the funders took in the study. If the funders had no role in your study, please state: "The funders had no role in study design, data collection and analysis, decision to publish, or preparation of the manuscript."

3) If any authors received a salary from any of your funders, please state which authors and which funders.

5) Please ensure to include the grant number in the Funding Information tab in your submission form. .

Note: If the reviewer comments include a recommendation to cite specific previously published works, please review and evaluate these publications to determine whether they are relevant and should be cited. There is no requirement to cite these works unless the editor has indicated otherwise.

**Reviewers' comments:**

Reviewer's Responses to Questions

**Comments to the Authors:**

**Please note that one review is uploaded as an attachment.**

Reviewer #1: Review uploaded as an attachment.

Reviewer #2: The manuscript describes the building and testing of software created by the authors for the rapid detection of latent tuberculosis from SERS data obtained from blood samples. The problem addressed is truly urgent, and providing open-source tools for such data processing is likely to be in high demand from the biophysical and medical communities.

However, there are some questions and suggestions to improve the integrity of this work. The main point is that the manuscript is written as a description of software and its testing (it is well done), focused on computer science specialists. On the other hand, the audience of the journal includes biologists and biomedical researchers who should be considered the target users of the proposed software. Thus, I recommend improving the text by addressing the following issues:

1. Although the origin of samples and their compounds subjected to the SERS study can be understood by reading Ref. 13, it would be better to characterize them shortly at the beginning of the manuscript; a potential user should clearly know the spectra of what should be uploaded into the program.

2. The preprocessing procedure requires further clarification in more detail: since the ML-based algorithms, which take into account not only peak positions but also their intensities, the meaning of relative arbitrary units must be clarified: Was the norming to the global maximum after the preliminary filtering and the baseline extraction applied? Or to some reference peak? Was a processed spectrum obtained shifted down to correspond to its minimal point to zero before normalization, etc? This information is crucial because Raman spectra obtained from biological samples are, as a rule, ‘dirty’; it is not a clear sequence of well-expressed peaks typical for chemistry. Whence, the preprocessing of such spectra may significantly affect the result. In particular, all spectra used for training and tests in this work were obtained with the same equipment and preprocessing procedure. But what happens when one will use a spectrum obtained in another lab? The standardization of input data must be uniform and well-described in detail.

3. Although the performance of the proposed software is well discussed from the computational point of view, it is still a ‘black box’ like most of the NN algorithms. But some additional biologico-spectral analysis of spectral features in charge of the response seems possible and will strengthen the work. In particular, it is interesting to discuss specific spectral regions that are most valuable for distinguishing between LTBI and non-TB cases (an example of such an analysis applied to another SERS-related problem can be found, e.g., in the work https://doi.org/10.7717/peerj.18830.) This can be done easily, either simply subdividing the whole spectrum into parts and processing shorter wavelength regions, or multiplying the spectrum by finite-length filters with different centering.

Reviewer #3: I'd like to thank the authors for their time in undertaking this important research and for the work they have carried out in writing this manuscript.

The manuscript describes TB-SERS Analyzer, a Python-based GUI tool integrating machine learning and a 1D-CNN for tuberculosis prediction from SERS spectral data. The software addresses a genuine gap where there is currently no dedicated tool for RS/SERS-based TB analysis; the four-module workflow is clearly described. However, several methodological concerns need to be addressed.

Major Concerns

The most significant weakness is the blind validation cohort of only four samples. While the concordance looks impressive in Table 2, four samples is wholly insufficient to draw meaningful conclusions about real-world diagnostic performance. A tool being proposed for clinical TB screening should be validated on a substantially larger independent cohort, ideally from a different collection site or patient population.

The retraining procedure raises a data leakage concern. The authors retrain the final 1D-CNN on the full 1,000-sample dataset using 80% training and 20% validation, but these are the same samples used for model selection and threshold tuning via LOOCV. Without a truly held-out external test set, reported performance metrics are likely optimistic.

The choice of IGRA as the reference standard is acknowledged as imperfect, but its implications deserve more discussion. IGRA cannot distinguish active TB from LTBI, has variable sensitivity in immunocompromised populations, and performs poorly in high-burden low-income settings, precisely where this tool is presumably intended to be deployed. The authors should discuss how IGRA-derived labels may have introduced systematic misclassification into the training data.

The confidence interval methodology is unconventional. Using the 33rd and 66th percentiles of predicted probabilities across TP/TN/FP/FN groups to define confidence tiers is not a standard approach and is not well-justified. The authors should either provide a statistical rationale or adopt a more established uncertainty quantification method.

Minor Concerns

The 79.75% accuracy at the 60:40 split, while the best-performing configuration, remains modest for a clinical screening tool. Some clinical context on what sensitivity/specificity trade-offs would be acceptable in TB screening practice would help readers interpret whether this level of performance is clinically useful.

The software is compiled only for Windows, despite the claim of platform independence. This is a practical barrier for various settings and should either be resolved or more clearly caveated.

The manuscript would benefit from a brief discussion of computational requirements, as the tool is positioned for resource-limited settings, yet no mention is made of minimum hardware specifications.

**********

**Have the authors made all data and (if applicable) computational code underlying the findings in their manuscript fully available?**

The PLOS Data policy requires authors to make all data and code underlying the findings described in their manuscript fully available without restriction, with rare exception (please refer to the Data Availability Statement in the manuscript PDF file). The data and code should be provided as part of the manuscript or its supporting information, or deposited to a public repository. For example, in addition to summary statistics, the data points behind means, medians and variance measures should be available. If there are restrictions on publicly sharing data or code —e.g. participant privacy or use of data from a third party—those must be specified.

Reviewer #1: **No:** Missing data, models and training scripts.

Reviewer #2: Yes

Reviewer #3: Yes

**********

PLOS authors have the option to publish the peer review history of their article (what does this mean?). If published, this will include your full peer review and any attached files.

If you choose “no”, your identity will remain anonymous but your review may still be made public.

**Do you want your identity to be public for this peer review?**  For information about this choice, including consent withdrawal, please see our Privacy Policy.

Reviewer #1: No

Reviewer #2: No

Reviewer #3: No

[NOTE: If reviewer comments were submitted as an attachment file, they will be attached to this email and accessible via the submission site. Please log into your account, locate the manuscript record, and check for the action link "View Attachments". If this link does not appear, there are no attachment files.]

**Figure resubmission:**

While revising your submission, we strongly recommend that you use PLOS’s NAAS tool (https://ngplosjournals.pagemajik.ai/artanalysis) to test your figure files. NAAS can convert your figure files to the TIFF file type and meet basic requirements (such as print size, resolution), or provide you with a report on issues that do not meet our requirements and that NAAS cannot fix.-->

After uploading your figures to PLOS’s NAAS tool - https://ngplosjournals.pagemajik.ai/artanalysis, NAAS will process the files provided and display the results in the "Uploaded Files" section of the page as the processing is complete. If the uploaded figures meet our requirements (or NAAS is able to fix the files to meet our requirements), the figure will be marked as "fixed" above. If NAAS is unable to fix the files, a red "failed" label will appear above. When NAAS has confirmed that the figure files meet our requirements, please download the file via the download option, and include these NAAS processed figure files when submitting your revised manuscript.

**Reproducibility:**

To enhance the reproducibility of your results, we recommend that authors of applicable studies deposit laboratory protocols in protocols.io, where a protocol can be assigned its own identifier (DOI) such that it can be cited independently in the future. Additionally, PLOS ONE offers an option to publish peer-reviewed clinical study protocols. Read more information on sharing protocols at https://plos.org/protocols?utm_medium=editorial-email&utm_source=authorletters&utm_campaign=protocols

Attachment Submitted filename: PCOMPBIOL-D-25-02316_review.pdf

# Author comment: Author response to Decision Letter 1

**DOI:** 10.1371/journal.pcbi.1014397.r002

16 Apr 2026

Attachment Submitted filename: Response to Reviewers.docx

# Peer review: Decision Letter 1

**DOI:** 10.1371/journal.pcbi.1014397.r003

**Authors:** Dominik Wodarz, Miguel Francisco de Rocha

2 Jun 2026

Dear Professor Faksri,

We are pleased to inform you that your manuscript 'TB-SERS Analyzer: Analysis tool for tuberculosis prediction based on Raman spectroscopy with machine learning and convolutional neural network' has been provisionally accepted for publication in PLOS Computational Biology.

Before your manuscript can be formally accepted you will need to complete some formatting changes, which you will receive in a follow up email. A member of our team will be in touch with a set of requests.

Please note that your manuscript will not be scheduled for publication until you have made the required changes, so a swift response is appreciated.

IMPORTANT: The editorial review process is now complete. PLOS will only permit corrections to spelling, formatting or significant scientific errors from this point onwards. Requests for major changes, or any which affect the scientific understanding of your work, will cause delays to the publication date of your manuscript.

Should you, your institution's press office or the journal office choose to press release your paper, you will automatically be opted out of early publication. We ask that you notify us now if you or your institution is planning to press release the article. All press must be co-ordinated with PLOS.

Thank you again for supporting Open Access publishing; we are looking forward to publishing your work in PLOS Computational Biology.

Best regards,

Miguel Francisco de Almeida Pereira de Rocha

Academic Editor

PLOS Computational Biology

Dominik Wodarz

Section Editor

PLOS Computational Biology

***********************************************************

Reviewer's Responses to Questions

**Comments to the Authors:**

**Please note here if the review is uploaded as an attachment.**

Reviewer #1: I am satisfied with the revisions.

Reviewer #2: This revision has clarified the issue related to the computational procedure and has provided additional discussion on the biological relevance and localization of the most significant spectral bands. Thus, I can recommend acceptance of this manuscript.

Reviewer #3: Thank you for addressing my concerns and comments!

**********

**Have the authors made all data and (if applicable) computational code underlying the findings in their manuscript fully available?**

The PLOS Data policy requires authors to make all data and code underlying the findings described in their manuscript fully available without restriction, with rare exception (please refer to the Data Availability Statement in the manuscript PDF file). The data and code should be provided as part of the manuscript or its supporting information, or deposited to a public repository. For example, in addition to summary statistics, the data points behind means, medians and variance measures should be available. If there are restrictions on publicly sharing data or code —e.g. participant privacy or use of data from a third party—those must be specified.

Reviewer #1: Yes

Reviewer #2: None

Reviewer #3: Yes

**********

PLOS authors have the option to publish the peer review history of their article (what does this mean?). If published, this will include your full peer review and any attached files.

If you choose “no”, your identity will remain anonymous but your review may still be made public.

**Do you want your identity to be public for this peer review?**  For information about this choice, including consent withdrawal, please see our Privacy Policy.

Reviewer #1: No

Reviewer #2: No

Reviewer #3: **Yes:** Michael Macgregor-Fairlie

# Editor report: Acceptance letter

**DOI:** 10.1371/journal.pcbi.1014397.r004

**Authors:** Dominik Wodarz, Miguel Francisco de Rocha

PCOMPBIOL-D-25-02316R1

TB-SERS Analyzer: Analysis tool for tuberculosis prediction based on Raman spectroscopy with machine learning and convolutional neural network

Dear Dr Faksri,

I am pleased to inform you that your manuscript has been formally accepted for publication in PLOS Computational Biology. Your manuscript is now with our production department and you will be notified of the publication date in due course.

The corresponding author will soon be receiving a typeset proof for review, to ensure errors have not been introduced during production. Please review the PDF proof of your manuscript carefully, as this is the last chance to correct any errors. Please note that major changes, or those which affect the scientific understanding of the work, will likely cause delays to the publication date of your manuscript.

Soon after your final files are uploaded, unless you have opted out, the early version of your manuscript will be published online. The date of the early version will be your article's publication date. The final article will be published to the same URL, and all versions of the paper will be accessible to readers.

For Research, Software, and Methods articles, you will receive an invoice from PLOS for your publication fee after your manuscript has reached the completed accept phase. If you receive an email requesting payment before acceptance or for any other service, this may be a phishing scheme. Learn how to identify phishing emails and protect your accounts at https://explore.plos.org/phishing.

Thank you again for supporting PLOS Computational Biology and open-access publishing. We are looking forward to publishing your work!

With kind regards,

Mahalakshmi Kannan

PLOS Computational Biology | Carlyle House, Carlyle Road, Cambridge CB4 3DN | United Kingdom ploscompbiol@plos.org | Phone +44 (0) 1223-442824 | ploscompbiol.org | @PLOSCompBiol
